# AKT Inhibitors: The Road Ahead to Computational Modeling-Guided Discovery

**DOI:** 10.3390/ijms22083944

**Published:** 2021-04-11

**Authors:** Amit Kumar Halder, M. Natália D. S. Cordeiro

**Affiliations:** LAQV@REQUIMTE/Faculty of Sciences, University of Porto, Rua do Campo Alegre, s/n, 4169-007 Porto, Portugal; amit.halder@fc.up.pt

**Keywords:** AKT inhibitors, multi-target QSAR models, pharmacophore-based mapping, molecular docking, molecular dynamics simulations

## Abstract

AKT, is a serine/threonine protein kinase comprising three isoforms—namely: AKT1, AKT2 and AKT3, whose inhibitors have been recognized as promising therapeutic targets for various human disorders, especially cancer. In this work, we report a systematic evaluation of multi-target Quantitative Structure-Activity Relationship (mt-QSAR) models to probe AKT’ inhibitory activity, based on different feature selection algorithms and machine learning tools. The best predictive linear and non-linear mt-QSAR models were found by the genetic algorithm-based linear discriminant analysis (GA-LDA) and gradient boosting (Xgboost) techniques, respectively, using a dataset containing 5523 inhibitors of the AKT isoforms assayed under various experimental conditions. The linear model highlighted the key structural attributes responsible for higher inhibitory activity whereas the non-linear model displayed an overall accuracy higher than 90%. Both these predictive models, generated through internal and external validation methods, were then used for screening the Asinex kinase inhibitor library to identify the most potential virtual hits as pan-AKT inhibitors. The virtual hits identified were then filtered by stepwise analyses based on reverse pharmacophore-mapping based prediction. Finally, results of molecular dynamics simulations were used to estimate the theoretical binding affinity of the selected virtual hits towards the three isoforms of enzyme AKT. Our computational findings thus provide important guidelines to facilitate the discovery of novel AKT inhibitors.

## 1. Introduction

AKT, also known as protein kinase B (PKB), is a serine/threonine-specific protein kinase that belongs to the AGC family of kinases. Three closely related isoforms of AKT occur in mammals, namely: AKT1 (PKBα), AKT2 (PKBβ) and AKT3 (PKBγ) [1,2]. All these AKT isoforms have a common structure comprised of three domains, i.e., a pleckstrin homology (PH) domain at the N terminus that binds to phosphatidylinositol-3-kinase (PI3K), a catalytic kinase domain with an ATP-binding site and a hydrophobic motif (HM) at the C-terminus [3]. A high degree of sequence homology is observed at the catalytic domain of AKT isoforms though the other two domains vary to a certain extent. AKT1 and AKT2 are expressed ubiquitously whereas AKT3 is found primarily in the brain, kidney, and heart. Being a key enzyme of the PI3K cascade, AKT plays a crucial role in the regulation of diverse cellular functions. Some major functions of AKT include cell proliferation, cell cycle progression, cell survival and inhibition of apoptosis through inactivation of more than 20 pro-apoptotic proteins [1,4,5,6]. AKT signaling is regularly impaired in several types of cancers and increased AKT activity has been detected in a number of aggressive malignancies. Therefore, these enzymes are considered as promising targets for the development of novel anticancer agents [7,8,9]. However, the scope of AKT inhibitors action is not just limited to the treatment of malignant diseases. Recent investigations suggested that AKT inhibitors might also be applied in the treatment of neurological diseases, diabetes, obesity, cardiovascular diseases, idiopathic pulmonary fibrosis, inflammatory and autoimmune diseases [6,10,11]. Multiple attempts have so far been made to develop AKT inhibitors as anticancer agents. GSK690693 was the first clinically tested AKT inhibitor that was followed by AZD5363, ipatasertib, afuresertib, uprosertib, MK-2206, etc. [12]. Currently, at least seven AKT inhibitors are in different stages of clinical trials [13]. Most of these clinically tested agents bind to the catalytic domain of the enzymes, and therefore these simultaneously inhibit all three isoforms of the AKT (i.e., they are pan-AKT-inhibitors). However, allosteric inhibitors were also developed in order to obtain more selectivity towards one or more AKT isoforms [14]. As an example, BAY1125976 is an allosteric inhibitor with higher specificity towards AKT1 and AKT2 isoforms. However, the advantage of isoform specific AKT inhibitors against pan-AKT inhibitors are yet to be established clinically [13,15].

Nevertheless, it has already been confirmed that bioactivity against all three isoforms should be taken into consideration while developing novel AKT inhibitors.

Machine learning-based (ML) tools have thus far been proved to be an extremely useful strategy for the design and discovery of therapeutically active agents [16,17,18]. In particular, these have been frequently employed in Quantitative Structure–Activity Relationships (QSAR) modeling to find structural requirements for higher active molecules and/or to predict activity of novel hit molecules [19,20,21]. ML tools have also been applied in multi-target QSAR (mt-QSAR) modeling for jointly predicting the bioactivity of compounds optimized under multiple biological targets and assay conditions [16,20,22,23,24,25,26]. Recently, we have launched the software code QSAR-Co for easy tackling of multi-target classification-based QSAR modeling efforts [22]. In QSAR-Co (available in https://sites.google.com/view/qsar-co (accessed on 8 February 2021)), linear mt-QSAR models are developed by the genetic algorithm based linear discriminant analysis (GA-LDA) whereas non-linear models are generated by the random forest (RF) technique. With the desire to extend its functionalities that can further help in understanding the scope and reliability of computational modeling-guided approaches, we examined here various feature selection algorithms and machine learning tools to build reliable mt-QSAR models for probing the inhibitory action of AKT enzyme isoforms. The best predictive linear and non-linear models were then used to screen a focused kinase library to obtain the most potential virtual hits that were further investigated by structure-based methods, such as pharmacophore-based prediction, docking and molecular dynamics (MD) simulation techniques. Even though several computational modeling works targeting AKT inhibitors have been reported so far, these were always focused only on one subtype of AKT pertaining to one experimental assay condition [27,28,29,30,31,32,33]. To the best of our knowledge, the current work is the first one to report multi-target computational modeling-guided discovery of inhibitors for all three AKT isoforms assayed under multiple experimental assay conditions.

## 2. Results and Discussion

### 2.1. Dataset Collection and Preparation

**Figure 1:**
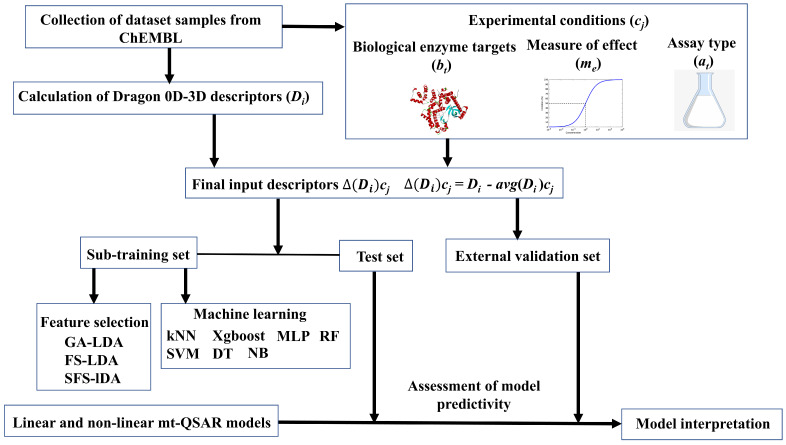
Flowchart showing the multi-target Quantitative Structure-Activity Relationships (mt-QSAR) work performed in the current work.

Dataset compounds were collected from the ChEMBL database (https://www.ebi.ac.uk/chembl/ (accessed on 1 June 2020)). Details of the dataset can be found in Supplementary Materials (SM1.xlsx). Each of these compounds has been tested against at least one of the three isoforms of AKT (i.e., AKT1, AKT2 and AKT3, BAO label: Single protein) and the corresponding activity evaluated according to either half-maximal inhibitory concentration (IC_50_) or binding affinity (Ki). Moreover, at least one of two assay techniques has been applied, i.e., either a binding assay (B) or a functional assay (F). After removal of duplicate data-points, a dataset containing 5523 samples was used for modeling the inhibitory activity, in which the latter was converted into a binary categorical response variable, *IAi*(*c_j_*), with values +1 (active) and −1 (inactive). Samples with IC_50_/K_i_ values ≤ 500 nM were considered as active [*IAi*(*c_j_*) = +1], otherwise they were considered as inactive [*IAi*(*c_j_*) = −1] [20,34]. Further, we adopted the Box-Jenkins moving average approach for handling the mt-QSAR modeling. Details of this computational modeling approach have been thoroughly discussed over the past and so we limit ourselves here to a brief outline [16,22,25,35]. According to the Box-Jenkins moving average approach, the input structural descriptors ($D_{i}$) of each compound are converted to deviation descriptors ($\Delta \left(D_{i}\right)c_{j}$ based on the experimental conditions $c_{j}$ (or ontology) these have been tested for. As referred to above, three different experimental elements are considered here for mt-QSAR modeling, i.e., the biological target ($b_{t}$: AKT1, AKT2, or AKT3), measure of effect ($m_{e}$: IC_50_ or Ki), and assay type ($a_{t}$: B or F). The final deviation descriptors $\Delta \left(D_{i}\right)c_{j}$ not only encode structural aspects of the compounds but also information related to the experimental conditions under which these have been assayed (i.e., $c_{j}$) [22,36,37]. Details regarding the calculation of input descriptors ($D_{i}$), data curation and dataset division schemes, as well as model development strategies are discussed in the Materials and Methods section. The mt-QSAR model development strategy is outlined in Figure 1.

### 2.2. Linear Interpretable Mt-QSAR Models

The dataset was first randomly divided into a training set containing 3867 data points (70% of the data) and a validation set containing 1656 data-points (30% of the data) using the random division technique of QSAR-Co tool [22]. A total of 5305 input descriptors ($D_{i}$) were calculated for the training set by employing the alvaDesc tool [38], and these descriptors were subsequently converted to 15,915 deviation descriptors with the help of QSAR-Co tool [22]. It must be noted here that all models were set up solely on the basis of the training set, which was further split into a sub-training set containing 2707 data points (70% of the training set) and a test set containing 1160 data points (30% of the training set) by applying the random division technique of QSAR-Co tool [22] for models’ development purposes. The predictivity of the built models was finally tested with the validation set. Therefore, the difference between the test set and validation set is that the test set datapoints participated in the calculation of deviation descriptors but datapoints of the validation set had no role on that.

Then, 2881 descriptors were identified to have an intercorrelation greater than 0.999 with other descriptors and after removing these descriptors the remaining 13,034 descriptors were considered for development of the linear models. However, the intercorrelations among the selected descriptors of the final models were critically examined.

Three different feature selection techniques, namely: genetic algorithm (GA), forward stepwise (FS) and sequential forward selection (SFS), were used one-by-one for the development of linear interpretable models. For each of the following linear discriminant analysis (LDA) models—i.e., GA-LDA, FS-LDA and SFS-LDA, a maximum of ten descriptors was allowed. The best linear models derived from the sub-training set, with descriptors selected by these feature selection techniques, are depicted in Table 1 along with the LDA statistical parameters.

As seen, the low Wilk’s lambda (λ) values and high chi-square (χ^2^), squared Mahalanobis distance (*D*^2^) and *F* values are indicative of the statistical significance of all three models developed. Among these models, the FS-LDA model is found to have the lowest λ value. Significantly, the goodness-of-fit of GA-LDA is very similar to that of the FS-LDA model. The degree of collinearity among the selected variables was also inspected, and the resultant cross-correlation matrices can be found in the Supplementary Materials (Tables S1–S3). The highest Pearson correlation coefficients (*r*) observed between two independent variables were 0.838, 0.614 and 0.742 for the GA-LDA, FS-LDA and SFS-LDA models, respectively. It must be pointed out here that we discarded all models generated with highly intercorrelated independent variables (*r* ≥ 0.85). That was the case, for example, of two initial SFS-LDA models that had to be discarded and then re-generated after removing one of the descriptors with *r* > 0.85.

The next step was to verify the uniqueness of the derived models, which can easily be done by applying the *Y*-based randomization technique [39]. In our previous works [16,20], the *Y*-based randomization was performed only by scrambling the response variable but here, we slightly modified this technique and named the new technique as *Yc* randomization. Generally, the *Y*-based randomization allows one to check if the linear model was not developed by chance. In conventional computational-guided modeling, the response variable is randomly shuffled *n* times to generate *n* number of randomized models, the statistical parameters of which are then compared to that of the original model [22,40]. However, in the Box-Jenkins based mt-QSAR, the experimental elements ($c_{j}$) participate also in the calculation of the final deviation descriptors. Therefore, these experimental elements should be randomized along with the response variables to assess the robustness of the models. In order to fulfil such criteria, both the responses $IA_{i}\left(c_{j}\right)$ and elements $c_{j}$ were shuffled 100 times to generate 100 different randomized datasets along with their deviation descriptors. The models developed subsequently using the same feature selection techniques were evaluated by computing the corresponding *λ* (*λ_r_*) values. The average of the latter values (*λ_rm_*) was then compared with the λ values obtained for the original models. The *λ_rm_* values obtained for the GA-LDA, FS-LDA and SFS-LDA randomized models (0.994, 0.996 and 0.992, respectively) were found to be much higher than the λ values obtained for the original models (0.414, 0.408 and 0.507, respectively), thus confirming the unique nature of the later models.

Let us now check the overall predictive ability of these linear models. To do so, statistical parameters such as the sensitivity, specificity, *F*-measure, accuracy and the Matthews correlation coefficient [41,42] values were carefully examined not only for the sub-training subset but also, to infer their external predictivity, firstly for the test set (*n* = 1160) and finally for the validation set (*n* = 1656). As seen in Table 2, all models display a high predictivity against the sub-training, test and validation sets. The overall predictivity of the GA-LDA model however supersedes that of both FS-LDA and SFS-LDA models, judging from the obtained accuracy values for such sets (88.2%, 89.6%, 88.2%, respectively). Interestingly, the overall predictivity of SFS-LDA model is similar to that of the GA-LDA model. Even though FS-LDA model had the highest goodness-of-fit (lowest *λ* value), it afforded a lower overall predictive power compared to that of the other two models.

**Figure 2:**
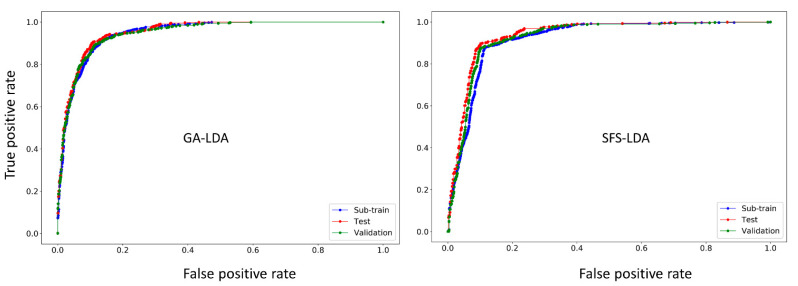
ROC curves for the two best linear models (GA-LDA and SFS-LDA).

Another way of confirming the classification ability of any LDA model is by means of the receiver operating characteristics (ROC) curve [43]. Figure 2 shows the ROC plots for the GA-LDA and SFS-LDA models. One can see that both these models are not random, but truly statistically significant classifiers, since the area under the ROC curves (ROC AUC scores calculated with Scikit-learn [44]) for all the sets are statistically higher (>0.88) than that of a random classifier model (ROC AUC score = 0.5).

To sum up, it can be inferred that the GA-LDA is the best linear model considering its goodness-of-fit as well as internal and external predictive ability. Moreover, when the sub-training set of GA-LDA model was subjected to a 10-fold cross-validation, an accuracy of 87.99% and MCC value of 0.752 were obtained indicating high internal predictivity of this model.

Yet, to establish the overall reliability of any mt-QSAR model, one should also access its applicability domain (AD). When the GA-LDA model was examined by means of the standardization-based AD approach [45], 64 data-points of the sub-training set, 20 data-points of test set and 49 data-points of the validation set are found to be possible structural outliers, meaning that for those predictions might not be reliable.

Further approval of this GA-LDA classification model should only be carried out after verifying if a consensus modeling approach might not yield a new model with higher predictivity. To accomplish this, the predicted response variables from three different LDA models were collected and the outcomes occurring more frequently were regarded as the consensus predicted activities. By comparing results from both models (Table 3), it was observed that the predictive power of the consensus LDA model is particularly similar but not higher than that of the original GA-LDA model. Therefore, the GA-LDA model is to be considered as the best linear interpretable model for the current dataset. However, considering the performance of the other LDA models, one can reach to the conclusion that the feature selection algorithms used here may simultaneously be applied for future mt-QSAR modeling of other datasets.

### 2.3. Interpretation of Molecular Descriptors

**Figure 3:**
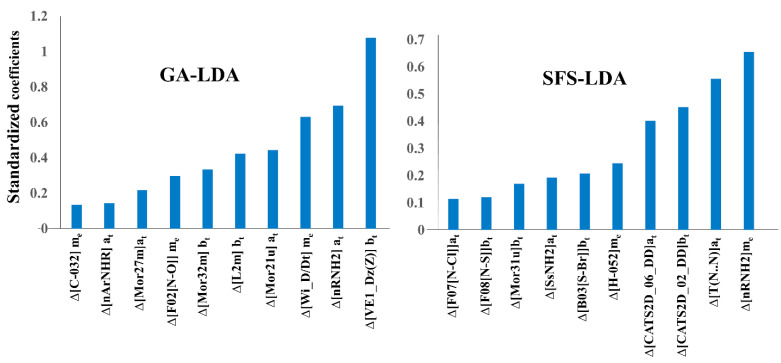
Absolute standardized coefficients vs. variables in the mt-QSAR models.

Undeniably, one of the primary advantages of linear models is the possibility of identifying the most crucial structural and physicochemical factors responsible for the higher activity of the compounds [46]. A description of all the descriptors appearing in the three linear models is given in Table 4. However, considering the higher statistical quality of both the GA-LDA and SFS-LDA models, our discussion will focus mainly on the descriptors appearing in these models. Since the relative contributions of the descriptors can only be understood by analyzing the absolute values of their standardized coefficients, these are shown in Figure 3 for the GA-LDA and SFS-LDA models.

Firstly, it is noteworthy that all the experimental elements considered in this work (i.e., *a_t_*, *m_e_*, and *b_t_*) consistently appeared in the final LDA models demonstrating their importance (Table 4). To set up the models, 30 distinct categories of descriptors (available in alvaDesc [38]) were considered but only 10 persisted. The latter pertained more frequently to 2D atom pairs (pairs of atoms at a given topological distance) and functional group counts descriptors, as well as to atom-centered fragments [49]. Importantly, these three categories of descriptors are easily interpretable. Nevertheless, 3D descriptors, such as 3D-Morse (3D-Molecular Representation of Structures based on Electronic diffraction) [50], WHIM (Weighted Holistic Invariant Molecular) [47], and CATS3D (Chemically Advanced Template Search 3D) [48], also appeared in the final models.

**Table 4:** Deviation descriptors of the mt-QSAR LDA models and their respective definitions.

Model	Deviation Descriptors	*c_j_*	Core Descriptor	Description	Descriptor Type ^a^
GA-LDA	$\Delta {\left[VE\_1Dz\left(Z\right)\right]}_{b_{t}}$	biological target	VE1_Dz(Z)	Coefficient sum of the last eigenvector (absolute values) from Barysz matrix weighted by atomic number	2D matrix-based
	$\Delta {\left(\mathrm{Mor}32m\right)}_{b_{t}}$	biological target	Mor32m	Signal 32/weighted by mass	3D-MoRSE
	$\Delta {\left(L2m\right)}_{b_{t}}$	biological target	L2m	2nd component size/weighted by mass	WHIM directional
	$\Delta {\left(C-032\right)}_{m_{e}}$	measure of effect	C−032	X--CX--X	Atom-centered fragments
	$\Delta {\left(F02\left[N-O\right]\right)}_{m_{e}}$	measure of effect	F02[N−O]	Frequency of N−O at topological distance 2	2D Atom Pairs
	$\Delta {\left(Wi\_D/Dt\right)}_{m_{e}}$	measure of effect	Wi_D/Dt	Wiener-like index from distance/detour matrix	2D matrix-based
	$\Delta {\left(\mathrm{nRNH}2\right)}_{a_{t}}$	assay type	nRNH2	Number of primary amines (aliphatic)	Functional group counts
	$\Delta {\left(\mathrm{nArNHR}\right)}_{a_{t}}$	assay type	nArNHR	Number of secondary amines (aromatic)	Functional group counts
	$\Delta {\left(\mathrm{Mor}27m\right)}_{a_{t}}$	assay type	Mor27m	Signal 27/weighted by mass	3D-MoRSE
	$\Delta {\left(\mathrm{Mor}21u\right)}_{a_{t}}$	assay type	Mor21u	Signal 21/unweighted	3D-MoRSE
FS-LDA	$\Delta {\left(D/Dtr05\right)}_{b_{t}}$	biological target	D/Dtr05	Distance/detour ring index of order 5	Ring
	$\Delta {\left(C-030\right)}_{b_{t}}$	biological target	C−030	X--CH--X	Atom-centered fragments
	$\Delta {\left(\mathrm{nCt}\right)}_{b_{t}}$	biological target	nCt	Number of total tertiary C(sp3)	Functional group counts
	$\Delta {\left(L2m\right)}_{b_{t}}$	biological target	L2m	2nd component size/weighted by mass	WHIM directional
	$\Delta {\left(\mathrm{CATS}3D\_18\_DL\right)}_{b_{t}}$	biological target	CATS3D_18_DL	Donor-Lipophilic BIN 18 (18–19 Å)	3D-CATS
	$\Delta {\left(\mathrm{CATS}3D\_10\_PL\right)}_{b_{t}}$	biological target	CATS3D_10_PL	Positive-Lipophilic BIN 10 (10–11 Å)	3D-CATS
	$\Delta {\left(\mathrm{nPyridines}\right)}_{m_{e}}$	measure of effect	nPyridines	Number of Pyridines	Functional group counts
	$\Delta {\left[T\left(N..O\right)\right]}_{m_{e}}$	measure of effect	T(N..O)	Sum of topological distances between N..O	2D Atom Pairs
	$\Delta {\left(\mathrm{CATS}3D\_07\_DA\right)}_{m_{e}}$	measure of effect	CATS3D_07_DA	Donor-Acceptor BIN 7 (7–8 Å)	3D-CATS
	$\Delta {\left(\mathrm{nRNH}2\right)}_{a_{t}}$	assay type	nRNH2	Number of primary amines (aliphatic)	Functional group counts
SFS-LDA	$\Delta {\left(F08\left[N-S\right]\right)}_{b_{t}}$	biological target	F08[N−S]	Frequency of N−S at topological distance 8	2D Atom Pairs
	$\Delta {\left(B03\left[S-Br\right]\right)}_{b_{t}}$	biological target	B03[S−Br]	Presence/absence of S−Br at topological distance 3	2D Atom Pairs
	$\Delta {\left(\mathrm{Mor}31u\right)}_{b_{t}}$	biological target	Mor31u	Signal 31/unweighted	3D-MoRSE
	$\Delta {\left(\mathrm{CATS}2D\_02\_DD\right)}_{b_{t}}$	biological target	CATS2D_02_DD	Donor-Donor at lag 2	2D-CATS
	$\Delta {\left(H-052\right)}_{m_{e}}$	measure of effect	H−052	H attached to C0(sp3) with 1X attached to next C	Atom-centered fragments
	$\Delta {\left(\mathrm{nRNH}2\right)}_{m_{e}}$	measure of effect	nRNH2	Number of primary amines (aliphatic)	Functional group counts
	$\Delta {\left(\mathrm{CATS}2D\_02\_DD\right)}_{b_{t}}$	assay type	T(N..N)	Sum of topological distances between N..N	2D Atom Pairs
	$\Delta {\left(F07\left[N-Cl\right]\right)}_{a_{t}}$	assay type	F07[N−Cl]	Frequency of N-Cl at topological distance 7	2D Atom Pairs
	$\Delta {\left(\mathrm{SsNH}2\right)}_{a_{t}}$	assay type	SsNH2	Sum of sNH2 E-states	Atom-type E-state indices
	$\Delta {\left(\mathrm{CATS}2D\_06\_DD\right)}_{a_{t}}$	assay type	CATS2D_06_DD	Donor-Donor at lag 6	2D-CATS

^a^ MoRSE: Molecular Representation of Structures based on Electronic diffraction; WHIM: Weighted Holistic Invariant Molecular [47]; CATS: Chemically Advanced Template Search [48].

As can be seen in Figure 3, the three most important descriptors of the GA-LDA model pertain to two topological indices (i.e., the graph-based descriptors $\Delta {\left[\mathrm{VE}\_1Dz\left(Z\right)\right]}_{b_{t}}$ and $\Delta {\left(Wi\_D/Dt\right)}_{m_{e}}$) and one constitutional descriptor ($\Delta {\left(\mathrm{nRNH}2\right)}_{a_{t}}$: counts of the number of primary amines). Other contributing descriptors of the model are geometrical descriptors either weighted by atomic masses or unweighted, these obviously containing information about the whole 3D-molecular structure of the compounds [47,49]. Interestingly, similar to the number of aliphatic amines, the number of aromatic amines ($\Delta {\left(\mathrm{nArNHR}\right)}_{a_{t}}$) is found also to be important in the GA-LDA model for triggering a higher biological activity, both being dependent on the experimental element assay type (*a_t_*). Except one 3D-MoRSE descriptor, all SFS-LDA descriptors are found as 2D descriptors. For instance, its most significant descriptor is $\Delta {\left(\mathrm{nRNH}2\right)}_{m_{e}}$, which along with the $\Delta {\left(\mathrm{nRNH}2\right)}_{a_{t}}$ descriptor of GA-LDA (which also appears in the FS-LDA model), reiterates the importance of aliphatic primary amines for achieving high activity against the AKT enzyme isoforms. Other important descriptors in this model are the frequency of atom pairs at particular topological distances, e.g., between two nitrogen atoms or sulfur and bromine atoms of the compounds [49]. Two mentions also are the two CATS2D descriptors [48] of the SFS-LDA model, i.e., descriptors $\Delta {\left(\mathrm{CATS}2D\_02\_DD\right)}_{b_{t}}$ and $\Delta {\left(\mathrm{CATS}2D\_06\_DD\right)}_{a_{t}}$, which embody a potential pharmacophore point on pairs of atoms. Both these involve two hydrogen bond donor groups (DD) in the compounds located at different topological distances.

Overall, it may thus be concluded that the presence as well as topological orientation of primary amines, amides and hydrogen bond donor groups in the molecules play crucial roles in promoting activity against the AKT isoforms. The topological distance between two nitrogen atoms, nitrogen and chlorine atoms, nitrogen and oxygen atoms, as well as sulfur and bromine atoms in the compounds appear to be the key factors for a higher AKT inhibition. At the same time, the importance of carbon atoms (e.g., $\Delta {\left(C-032\right)}_{m_{e}}$ and $\Delta {\left(H-052\right)}_{m_{e}}$), topological graph-based descriptors and geometrical descriptors (particularly 3D-MoRSE ones) is revealed. All these descriptors are found to be significant in ascertaining the biological activity of the compounds against AKT enzymes.

### 2.4. Non-Linear Predictive Mt-QSAR Models

Herein, descriptors with variances less than 0.001 and inter-correlations higher than 0.95 were removed, leaving 3342 deviation descriptors for setting up various non-linear mt-QSAR models, based on the ML tools: Xgboost [51], (RF) [52], *k*NN [53], RBF-SVC [54], MLP [55], DT [56], and NB [57]. Since the statistical quality of the resultant non-linear models substantially depends on the ML parameter settings [58], the latter were optimized by hyperparameter tuning [59]. A 10-fold cross-validation (CV) grid search scheme was employed to optimize the parameters and to achieve the best possible model based upon the sub-training set.

Table 5 shows the parameter values that were optimized through hyperparameter tuning as well as the final optimized values of these parameters for each of the machine learning tools applied. Furthermore, Table 5 also depicts the 10-fold CV accuracies obtained when the machine learning tools with optimized parameter values were fitted with the sub-training set.

It is evident that the classification ability of these machine learning tools varies to a considerable extent, but those achieving the better ones are the DT, RF and Xgboost tools. In fact, the MLP model demonstrates moderate internal predictivity, and the classifications produced by *k*NN, SVC and NB suggest an extremely poor internal predictivity of these tools.

The RF and Xgboost tools produced a 10-fold CV accuracy on the sub-training set of 91.02% and 91.54%, respectively, indicating that both resultant models display high and almost similar internal predictivity. These two models were then chosen to further investigate their external predictivity on the test and validation sets. Table 6 shows the overall predictivity of the RF and Xgboost models, whereas their ROC plots are displayed in Figure S1 of the Supplementary Materials.

As expected, the internal and external predictivities of the RF and Xgboost based non-linear models are noticeably higher than those of the linear models (see Table 2 and Table 3). On the basis of their overall predictivity (cf. accuracy values and MCC scores for all sets in Table 6), the Xgboost model stands for the best non-linear model.

One aspect that warrants explicit attention is to inspect how the two best-derived models (i.e., GA-LDA and Xgboost) manage to classify the datapoints pertaining to different experimental elements *c_j_* (Table 7). In general, the datasets applied in mt-QSAR computational modeling encompass a large variation in the number of data-points vis-à-vis the various experimental elements. As expected, the same situation happens in the current dataset. Still, the non-linear Xgboost model is unaffected by that since it affords high accuracies irrespectively of the experimental element or validation set. The GA-LDA model, with less overall predictivity than the Xgboost model, shows also high accuracies in case of the test set. Nevertheless, it reaches low accuracy values against some experimental conditions (e.g., for *c_j_* = 4 and 7). Yet, if both these models are considered simultaneously, there is apparently a greater chance of finding more accurate predictions.

### 2.5. Virtual Screening

**Figure 4:**
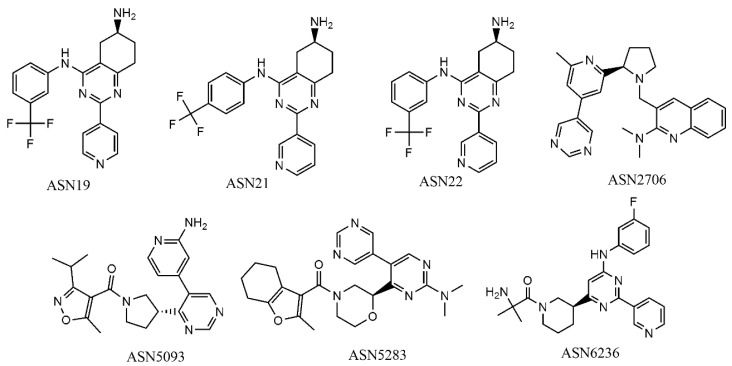
Structures of the most potential virtual hits obtained with the GA-LDA and Xgboost models.

In order to describe how the developed mt-QSAR models perform on identifying virtual hits, we employed the GA-LDA and Xgboost models for screening the focused library ***Asinex kinase*** (http://www.asinex.com/focus_kinases/, accessed on 17 August 2020), which comprises 6538 compounds. Details about this dataset can be found in Supplementary Materials (SM2.xlsx). Similarly, the descriptors of all such compounds were calculated by the alvaDesc tool [38]. In the modeling dataset used here, we found 10 unique experimental elements *c_j_* depending on the *m_e_*, *a_t_* and *b_t_* conditions (cf. Table 7). Each compound of the Asinex kinase inhibitor library was also assigned to those conditions, and a virtual dataset containing 65,380 samples was then prepared. Afterwards, the deviation descriptors of each of these samples were calculated using the QSAR-Co tool [22]. Initially the GA-LDA model was used for screening this virtual dataset, and 28 compounds were predicted as active (*IA_i_(c_j_*) = +1) against at least 7 out of 10 experimental elements. The predictivity of these 28 compounds was then tested with the best non-linear model (i.e., the Xgboost model) and seven compounds were predicted to be active against at least 6 experimental elements *c_j_*. These seven compounds (Figure 4) are thus to be considered as the most potential virtual hits according to the current computational multi-target modeling. Note that, for screening the dataset, we mainly relied on the linear model since it is likely to be less susceptible to overfitting than the non-linear model.

Table 8 displays the number of experimental conditions pertaining to each virtual hit picked by the GA-LDA and Xgboost models, whereas details of these experimental conditions are provided in Supplementary Materials (Table S4). It is observed that each of these virtual hits are predicted to be active against all three AKT isoforms in one or more experimental assay conditions (defined by the combinations of *a_t_* and *b_t_*).

### 2.6. Pharmacophore Based Biological Target Identification

After identifying seven virtual hits from multi-target computational-guided modeling, we have decided to implement a filtering scheme based on reverse pharmacophore mapping. The latter will allow pre-filtering such hits that best fulfil simple geometric and chemical functionality requirements to trigger a biological response, before embarking on more computationally demanding approaches. For this, each of these virtual hits were investigated with the PharmMapper webserver (http://www.lilab-ecust.cn/pharmmapper/index.html, accessed on 4 January 2021)) [60,61] to identify their possible human macromolecular targets. The results of PharmMapeer based predictions are outlined in Table 9. Details about the fitting of the virtual hits with the obtained structure-based pharmacophores are provided in the Supplementary Materials (Figures S2 and S3).

Interestingly, except Asn5283 and Asn2706, all virtual hits are mapped with the structure-based pharmacophores coming from both human AKT1 (PDB ID: 3CQU) and human AKT2 (PDB ID: 2UW9). Note that the PharmMapper predictions are based on the protein structures available in the Protein Data Bank (PDB) and so far, no complete structure of AKT3 is available. Therefore, these results mean that multiple AKT isoforms may be possible biological targets for these five virtual hits, which are Asn0019, Asn0021, Asn0022, Asn5093 and Asn6236. Furthermore, all these five virtual hits are fitted with structure-based pharmacophores generated at the catalytic sites of these enzyme isoforms. We randomly selected five Asinex kinase inhibitor library compounds (names and structures are provided in SM2.xlsx of Supplementary Materials) that were predicted to be inactive in the QSAR based virtual screening and none of these compounds was mapped with structure-based pharmacophores pertaining to both AKT1 and AKT2. Based upon the current pharmacophore analysis, we removed Asn5283 and Asn2706, and decide to further investigate the remaining six virtual hits by other more complicated structure-based analysis techniques.

### 2.7. Structure-Based Prediction of the Virtual Hits

Even though pharmacophore mapping insinuated that selected virtual hits may interact with multiple AKT enzyme isoforms, considering high flexibilities of kinase enzymes, it may not be able to predict the stabilities ligand-receptor complexes. Therefore, we decided to go a step forward and carry out molecular dynamics (MD) simulations to understand the dynamic behavior of the virtual hits within the AKT enzyme isoforms. The five virtual hits predicted by both the mt-QSAR modeling and the pharmacophore-based mapping (PharmMapper) were initially docked into the isoforms AKT1 (PDB ID:4GV1) and AKT2 (PDB ID: 1O6K), as well as into an AKT3 homology model. For this, two molecular docking tools were employed, namely: (a) Autodock Vina [62] and (b) Autodock 4.2 [63]. The homology modeling of AKT3 was carried out using the SWISS-MODEL webserver (swissmodel.expasy.org) [64,65]. Details about the procedures of homology modeling used as well as the validation of the model are described in the Materials and Methods section.

Since multiple binding sites exist in these AKT enzyme isoforms, we initially conducted a blind docking experiment for the virtual hits with the help of Autodock Vina. To do so, a grid box of 100 Å × 100 Å × 100 Å dimensions was centered on each of such macromolecules. The blind docking (performed with an exhaustiveness of 45) indicated that all the virtual hits may preferably bind to the catalytic domain of the enzyme isoforms. Thereafter, an Autodock based docking was performed for all the virtual hits by setting a grid box of 50 Å × 50 Å × 50 Å dimensions, with a grid spacing of 0.375 Å, centered on the catalytic residue of Asp292, Asp293, and Asp289 for AKT1, AKT2, and AKT3, respectively. The pan-AKT inhibitor GSK690693 was employed as a reference compound in this Autodock docking experiment. The results of both docking experiments performed with Autodock Vina and Autodock 4.2 are provided in Table S5 of Supplementary Materials.

These MD simulations were carried out with docked (Autodock-based) complexes of these compounds for 50 ns. The docked complexes of some selected virtual hits (i.e., the starting ligand-protein complexes used in the MD simulations) are provided in the Supplementary Materials. The root-mean-square-deviation (RMSD) plots of the backbone atoms of the receptor-ligand complexes, ligand RMSD plots, the root-mean-square-fluctuation (RMSF) plots of these protein structures, and the radius of gyration’ plots are presented in the Supplementary Materials (Figures S4–S6). A close look at these plots reveals an adequate dynamic stability as well as compactness of the ligand-macromolecule complexes. However, our major goal was to estimate the binding free energies of these ligand-protein complexes, which was computed by the molecular mechanics-generalized Born surface area (MM-GBSA) approach.

As observed from the results in Table 10, Asn0019 depicts maximum theoretical binding energy values against three different enzyme isoforms of AKT among five virtual hits. The theoretical binding free energy values of Asn0019 against AKT2 and AKT3 enzymes were comparable to those of the reference compound GSK690693. Even though Asn5093 depicted slightly increased theoretical ∆G_bind_ values against AKT1 and AKT2 as compared to Asn0019 (as well as Asn0021 and Asn0022), its binding energy was found to be reduced against AKT3 enzyme. The Asn0019 may be projected as the most promising virtual hits based on its average theoretical ∆G_bind_ value.

**Figure 5:**
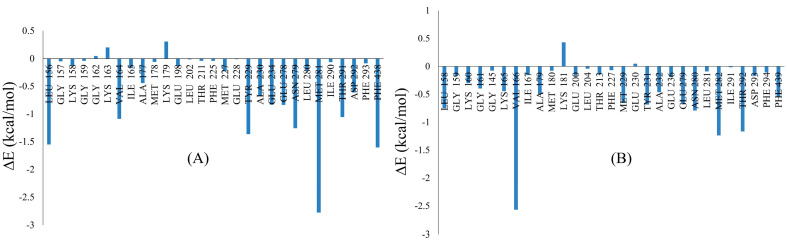
Total energy obtained from per-residue decomposition analysis of Asn0019 in (**A**) AKT1 and (**B**) AKT2 enzymes.

Per residue decomposition analysis of Asn0019 was performed to understand the binding interactions of this virtual hit against X-ray crystal structures AKT isoforms (i.e., AKT1 and AKT2). Total energy values obtained from important binding site residues are depicted in Figure 5. It is observed that interactions of Asn0019 with Leu156/158, Val164/166, Ala177/179, Lys179/181, Tyr229/231, Ala230/232, Glu278/279, Asn279/280, Met281/282, Thr291/292 and Phe438/439 of AKT1 and AKT2 enzymes may play crucial roles in determining the binding affinities of this molecule in these proteins.

## 3. Materials and Methods

### 3.1. Descriptor Calculation

The SMILES structures of the compounds were converted to 2D structures using the MarvinView software (https://docs.chemaxon.com/display/docs/MarvinView, accessed on 6 July 2020). Such structures were subsequently standardized by the ChemAxon Standardizer tool using the following options: strip salts, aromatize, clean 3D, tautomerize, neutralize and add explicit hydrogens [66]. The initial descriptors were calculated by alvaDesc tool (https://www.alvascience.com/alvadesc/, accessed on 6 July 2020) [38] in the OCHEM web-platform [67]. Geometry optimizations of the compounds were performed with the Corina software [68] under OCHEM [67]. Calculation of the deviation descriptors (Δ(*D_i_*)*c_j_*) from the initial descriptors (*D_i_*) and experimental elements (*c_j_*) were carried out using the QSAR-Co tool [22].

### 3.2. Development of Linear Interpretable Models

The linear models were based on linear discriminant analysis (LDA) models and three different feature selection algorithms, i.e., (a) genetic algorithm (GA), (b) forward stepwise (FS), and (c) sequential forward selection (SFS). For the first one, the resultant GA-LDA model was set up with the help of the QSAR-Co tool [22]. Details of the GA selection procedure have been extensively reported in the past [69]. Briefly, GA is based on the evolution of a population of randomly generated models. Firstly, parent models are chosen, and these are then subjected to random “cross-over” and ”mutation” processes to produce child models, which are then used to check their fitness scores. The new generated models with the highest fitness scores are then forwarded to a next iteration. The algorithm terminates either when the maximum number of allowed population models is reached or when no improvement regarding the fitness score is observed for the subsequent 10 population generations. The parameters employed here for setting up the GA-LDA model with QSAR-Co were: (i) total number of iterations/generations: 100, (ii) equation length: 10 (fixed), (iii) mutation probability: 0.3, (iv) initial number of equations generated: 100, and (v) number of equations selected in each generation: 30. Forward stepwise (FS) is a very popular feature selection algorithm in which the independent descriptors are included in the model stepwise depending on a specific statistical parameter. In this work, the FS-LDA model was set up with a python program where the features are selected and included in the model stepwise by the corresponding *p*-values of the Fischer statistic [69]. Initially, criteria for the forward selection (i.e., *p*-value to enter) as well as for the backward elimination (*p*-value to remove) have to be set. The descriptor with the lowest *p*-value is first included and subsequently other descriptors are included in the model based on their *p*-values only if the criterion for forward selection is met. However, if the *p*-value of a descriptor included in the model is found to be greater than the ”*p*-value to remove”, it is eliminated from the model. In the current work, both *p*-values to enter and to remove were fixed at 0.05. The final LDA models were developed and were subsequently validated using the *LinearDiscriminantAnalysis* function of Scikit-learn [44]. The python code used for FS-LDA model development is provided in the Supplementary Materials (file SM1.py). The last feature selection algorithm is based on a sequential forward selection which adds features into an empty set until the performance of the model is not improved either by addition of another feature or by reaching the maximum allowed number of features [70]. Similar to FS, this procedure is also a greedy search algorithm where the best subsets of descriptors are selected stepwise and the model performance is justified by the user-specific statistical measure. In this work, the python based SequentialFeatureSelector algorithm of the “mlxtend” library (http://rasbt.github.io/mlxtend/, accessed on 27 July 2020) was applied to setup the resultant SFS-LDA model. The parameters used for that purpose were: (i) maximum number of features: 10, (ii) forward: True, (iii) floating: True, (iv) scoring: accuracy, and (v) cross-validation (cv) = 0. Thereby, accuracy was employed as the user-specific statistical measure for feature selection, in contrast to FS where the *p*-value was used, and no cross-validation was performed during feature selection.

Several diagnostic statistical indices were employed for assessing our model equations, in terms of the criteria goodness-of-fit and goodness-of-prediction. Measures of goodness-of-fit of the LDA models based on the sub-training set were estimated by standard indices such as the Wilks’ lambda (λ) [71], chi-squared (χ^2^), the square of Mahalanobis distance (*D*^2^), the Fisher’s statistic index (*F*), and the corresponding *p*-value (*p*) [41]. Measures of goodness-of-prediction for both linear and non-linear models were estimated by computing the following statistical measures for the sub-training, test and external validation sets: sensitivity—correct classification of the active cases, specificity—correct classification of inactive cases, accuracy—overall correct classification, *F*-measure, and Matthews correlation coefficient (MCC) [41,42].

### 3.3. Non-Linear Model Development

Seven different machine learning techniques were used for setting up the non-linear models, namely: (a) gradient boost classifier (Xgboost) [51], (b) Random Forests (RF) [52], (c) *k*-Nearest Neighbors (*k*NN) [53], (d) Radial Basis Function based Support Vector Classifier (RBF-SVC) [54], (e) Multilayer Perceptron (MLP) neural-networks [55], (f) Decision Trees (DT) [56], and (g) Bernoulli Naïve Bayes (NB) [57]. The python 2 based Xgboost (version 1.0.0) algorithm (https://xgboost.readthedocs.io/en/latest/, accessed on 26 October 2020) was used for developing the Xgboost models. Regarding the RF ML, that was implemented with the help of the QSAR-Co tool [22], using the following parameters: (i) each bag size: 100, (ii) maximum depth: 0 (unlimited), (iii) number of randomly chosen features: 0 [i.e., *n* = int(log2(#Predictors) + 1)], and (iv) number of iterations: 100.

All other machine learning techniques were applied by resorting to the respective Scikit-learn machine learning packages [44]. The model development parameters for each of these techniques were determined by hyperparameter tuning as implemented in GridSearchCV of Scikit-learn [58]. During hyperparameter optimization, a 10-fold cross-validation (10-fold CV) was performed with the sub-training set to identify the best model estimators. The 10-fold CV accuracies obtained from the different machine learning techniques were compared to find the highly predictive classifiers. Finally, the external predictivity of these classifiers were estimated with the test and the validation sets.

### 3.4. PharmMapper Based Prediction of Biological Targets

The freely-accessed PharmMapper (version 2017) web server (http://www.lilab-ecust.cn/pharmmapper/, accessed on 4 January 2021) searches for the best mapping poses of the given molecules against structure-based pharmacophore models generated with all targets of PharmTargetDB [60,61]. PharmMapper applies a large database of receptor-based pharmacophores (>7000 pharmacophore models based on 1627 drug targets, 459 of which are human protein targets.) to find possible macromolecular targets for the input ligands. It implements Cavity (version 1.1) program in order to identify the binding sites on the surface of a protein structure and to rank these subsequently as per their druggability scores. The receptor-based pharmacophore modelling was then performed using Pocket (version 4.0) program for extracting the pharmacophore features within these druggable cavities.

In this work, the virtual hits obtained from multi-target QSAR computational modeling were subjected to PharmMapper based predictions of biological targets. A maximum number of 1000 conformers were generated during the search of pharmacophore fitting on human biological targets and the top 200 targets based on the fit scores were analyzed.

### 3.5. Homology Modeling

The homology model of AKT3 was set up with the help of the SWISS-MODEL webserver [64,65]. The FASTA sequence of human AKT3 was retrieved from Uniprot (Uniprot ID: Q9Y243) and we used the sequence 141–479 for homology modeling. The FASTA sequence was uploaded to the SWISS-MODEL server to search for templates and then, ranging from 50 templates to 10 templates were selected for generating the model on the basis of higher GMQE values and also on their diversity. Each of the homology models was analyzed by the ‘structural assessment’ option available in this webserver. Among these models, one model developed with the template of 6CCY.1.A (sequence identity: 83.18%) was found to have the lowest MolProbity value (=1.02), calculated from http://molprobity.biochem.duke.edu/, accessed on 11 January 2021). This model, which showed a Qualitative Model Energy Analysis (QMEAN) value of −1.84, was selected for further structural modifications. From the low MolProbity value, it is ensured that the homology model is of good structural quality, however, the model showed some complications mainly due to its Clashscore (all atoms) of 0.37, the presence of 5 Ramachandran outliers, as well as the presence of 26 bad angles. Therefore, the UCSF Chimera software [72] was utilized for structural modifications of this homology model. The latter included step-by-step loop refinement, rotamer adjustment and structural minimization of the selected residues. Then, for each modification step, the quality of the model was checked in the Molprobilty webserver (http://molprobity.biochem.duke.edu/, accessed on 11 January 2021) [64]. Ultimately, the modified homology model was found to have an even lower Molprobilty score of 0.87, indicating that the initially developed model was clear structurally improved. In this final model, the Clashscore (all atoms) reduced to zero and the number of outliers to one, as well as the number of bad angles lowered to two. The final model also depicted an improved Global Model Quality Estimation (GMQE) value of −1.61. Alignment of Q9Y243 with 6CCY.1.A, validation parameters as well as Ramachandran plots of the final homology model are shown in the Supplementary Materials (Figures S7 and S8).

### 3.6. Molecular Docking

The docking experiments with the X-ray crystal structures and homology model were performed with Autodock Vina (version 1.1.2., The Scripps Research Institute, La Jolla, CA, USA) [62] and Autodock 4.2 (The Scripps Research Institute, La Jolla, CA, USA) [63]. During preparation of the macromolecules, water molecules and the peptide substrate obtained from the PDB were removed. Other necessary details about the methodology followed on such docking experiments are the same as described earlier [20].

### 3.7. Molecular Dynamics Simulations

All MD simulations were carried out using the software package AMBER 12 [73,74]. Initially, the protonation states of the amino acid residues of each protein were fixed at pH = 7.0. These protonation states were attained by the PDB2PQR server (http://server.poissonboltzmann.org/pdb2pqr, accessed on 12 January 2021), using the AMBER forcefield and output naming scheme [75]. The ff99SB and general AMBER forcefield (GAFF) were applied for describing the protein-inhibitor and inhibitor-solvent interactions, respectively [76,77]. The optimization of ligands was carried out with the help of Leap in Antechamber, by performing MD simulations of the hydrated complexes centered in a cubic box of edge length of 8 Å, and applying the forcefields GAFF, ff99SB, and TIP3P for water molecules [78]. Subsequently, the negative charges of the complex systems were neutralized. The SHAKE algorithm was used to constrain all bonds related to hydrogen atoms, whereas the Partial Mesh Ewald (PME) method was employed to handle long range electrostatic forces (using a cutoff of 12 Å). Energy minimization of the complexes was performed in two stages. In the first stage, only the ions and water molecules were allowed to relax during 1000 steps of the steepest descent method and during 1000 steps of the conjugate gradient algorithm using a restrained force of 500 kcal/mol on the solute. In the second stage, the whole system was relaxed during 5000 minimization steps, i.e., 2500 steps of steepest decent minimization followed by 2500 of conjugated gradient. The minimized systems were then gradually heated up from 0 to 300 K (50 K in each step) with a weak harmonic restraint of 10 kcal/mol to keep the solute fixed for 200 ps. Subsequently, a 2-ns equilibration on the *NpT* ensemble was performed, with pressure kept fixed at 1 bar and temperature at 300 K. Finally, the 50-ns MD simulations without restrictions were run with constant temperature (*T* = 300 K) and constant pressure (*p* = 1 bar). After completion of such simulations, various post-dynamic analyses were carried out with PTRAJ and CPPTRAJ implemented in the AMBER package [79]. The graphs were plotted using the QTGRACE tool (https://sourceforge.net/projects/qtgrace/files/, accessed on 1 February 2021). Molecular Mechanics Generalized Born Surface Area (MM-GBSA) based binding free energies of the ligands were calculated using MMPBSA.py program in AMBER [80,81]. One hundred snapshots were taken from the last 10 ns of MD trajectory.

The binding energy calculation is represented as the following equation:

(1) $$∆G=∆E_{ele}+∆E_{vdW}+∆G_{pol}+∆G_{nonpolar}-T∆S$$

∆*E_ele_* and ∆*E_vdW_* are electrostatic and van der Waals interactions between the ligands and the proteins (in gas phase), respectively. The polar solvation free energy (∆*G_pol_*) accounts for the polar interactions with the solvent molecules whereas the ∆*G_nonpolar_* term represents non-polar solvation free energy, which is obtained from the equation $∆G_{nonpolar}=\gamma SASA+\beta$. The the solvent accessible surface area is represented by the term SASA. The surface tension proportionality constant (γ) and the free energy of nonpolar solvation of a point solute (β), were set to 0.00542 kcal mol^−1^ Å^−2^ and 0 kcal mol^−1^, respectively. The entropic contribution (*T*∆*S*) is not calculated because apart from being computationally expensive (especially for large macromolecular complexes), it has been reported to be less accurate [80,81].

The energy contributions of the close contact amino acid residues into the total binding free energies were computed using MM-GBSA per residue free energy decomposition method with Amber 12 MM-GBSA module [73,74,82,83]. All energy components (van der Waals, electrostatic, polar solvation, and nonpolar solvation contributions) were calculated using 200 snapshots extracted from the last 10 ns MD trajectories.

## 4. Conclusions

The latest advances in machine learning tools, coupled with the availability of ever-larger data sets, brought about a fresh wave for faster and less complicated computational-guided drug discovery efforts [84,85,86]. In this work, we could assemble a large dataset containing 5523 inhibitors of the three AKT isoforms, assayed under a variety of experimental conditions. With the desire to build reliable predictive multi-target QSAR classification models for probing the inhibitory activity from such data, we examined the use of various machine learning tools along with several feature selection algorithms. Considering that machine learning is a powerful mean for finding drug like leads [18], the best linear and non-linear mt-QSAR models were finally used for screening a focused kinase library to identify virtual hits as potential pan-AKT inhibitors. The results obtained were finally post-processed by structure-based approaches.

With regard to the mt-QSAR modeling, the combination of LDA with feature selection algorithms such as FS, SFS, or GA was found to produce classification models exhibiting very good accuracy (>87%), as well as internal and external predictivity. Nevertheless, the GA feature selection algorithm yielded the best predictive linear model, even though in a not so straightforward and less time-consuming way as the other two algorithms. More significantly, these linear models aided us in understanding the most crucial structural and/or physicochemical properties required for higher AKT inhibition. At the same time, the classification ability of the seven different ML-based mt-QSAR models were found to vary to a considerable extent. The Xgboost technique produced the most predictive non-linear mt-QSAR model (accuracy > 90%), but its overall predictivity was similar to that of the RF model. This leads us to suppose that tree-based modeling techniques are superior to other machine learning ones for multi-target computational modeling. Yet, more investigations are needed to confirm that supposition.

To judge how the best linear and nonlinear mt-QSAR models perform on identifying virtual hits with activity against AKT inhibitors, we used them to screen the Asinex kinase inhibitor library. The obtained virtual hits were further evaluated by structure-based pharmacophore modeling, molecular docking and MD simulations studies. Worth mentioning here that in case of multi-target modeling, structure-based approaches become more problematic when one or more biological targets are not sufficiently characterized. Indeed, this was the case for the AKT3 isoform, the X-ray crystal structure of which is yet to be reported, and thereby a reliable homology model had to be derived. The pharmacophore-mapping target-identification search led to results reinforcing the former mt-QSAR based predictions. Further, the results obtained in the following MD simulations allowed us to put forward Asn0019 as the most potent virtual hit for the inhibition of all AKT isoforms.

To conclude, the information gathered and the derived mt-QSAR computational models provide important guidelines for the discovery of novel AKT inhibitors. What is more, such models are not limited only to pan-inhibitors but can also be applied to identify inhibitors that have selectivity towards one or two AKT isoforms.

## Figures and Tables

**Table 1:** Goodness-of-fit of the linear models produced by different feature selection algorithms.

Method	Model	λ	χ^2^	*D* ^2^	*p*	*F* (10,2696)
GA-LDA	$\begin{array}{ll} IA_{i}\left(c_{j}\right)= & +1.766\Delta {\left[VE1\_Dz\left(Z\right)\right]}_{b_{t}}+1.399\Delta {\left(Mor32m\right)}_{b_{t}}+0.173\Delta {\left(L2m\right)}_{b_{t}}\\ & -0.464\Delta {\left(C-032\right)}_{m_{e}}-0.280\Delta {\left(F02\left[N-O\right]\right)}_{m_{e}}-0.004\Delta {\left(Wi\_D/Dt\right)}_{m_{e}}\\ & +1.186\Delta {\left(nRNH2\right)}_{a_{t}}+0.768\Delta {\left(Mor27m\right)}_{a_{t}}+0.549\Delta {\left(Mor21u\right)}_{a_{t}}\\ & -0.323\Delta {\left(nArNHR\right)}_{a_{t}}+1.532 \end{array}$	0.414	2381.34	5.89	<10^−16^	374.53
FS-LDA	$\begin{array}{ll} IA_{i}\left(c_{j}\right)= & +2.043\Delta {\left(C-030\right)}_{b_{t}}+0.953\Delta {\left(\mathrm{nCt}\right)}_{b_{t}}+0.309\Delta {\left(L2m\right)}_{b_{t}}+0.013\Delta {\left(D/Dtr05\right)}_{b_{t}}\\ & -0.589\Delta {\left(\mathrm{CATS}3D\_18\_DL\right)}_{b_{t}}-0.460\Delta {\left(\mathrm{CATS}3D\_10\_PL\right)}_{b_{t}}\\ & +1.016\Delta {\left(\mathrm{nPyridines}\right)}_{m_{e}}+0.481\Delta {\left(\mathrm{CATS}3D\_07\_DA\right)}_{m_{e}}-0.007\Delta [{\left(T\left(N..O\right)\right]}_{m_{e}}\\ & -3.545\Delta {\left(\mathrm{nRHNH}2\right)}_{a_{t}}+2.545 \end{array}$	0.408	2420.04	6.156	<10^−16^	391.07
SFS-LDA	$\begin{array}{ll} IA_{i}\left(c_{j}\right)= & +0.954\Delta {\left(F08\left[N-S\right]\right)}_{b_{t}}+0.851\Delta {\left(\mathrm{Mor}31u\right)}_{b_{t}}-1.081\Delta {\left(\mathrm{CATS}2D\_02\_DD\right)}_{b_{t}}\\ & -3.280\Delta {\left(B03\left[S-Br\right]\right)}_{b_{t}}+2.494\Delta {\left(\mathrm{nRNH}2\right)}_{m_{e}}+0.177\Delta {\left(H-05\right)}_{m_{e}}\\ & +0.567\Delta {\left(F07\left[N-Cl\right]\right)}_{a_{t}}+0.099\Delta {\left(\mathrm{SsNH}2\right)}_{a_{t}}+0.005\Delta {\left[T\left(N..N\right)\right]}_{a_{t}}\\ & -1.419\Delta {\left(\mathrm{CATS}2D\_06\_DD\right)}_{a_{t}}+1.543 \end{array}$	0.507	1831.98	4.120	<10^−16^	261.77

**Table 2:** Overall performance of the final linear models.

Classification ^a^	GA-LDA			FS-LDA			SFS-LDA		
	Sub-Training	Test	Validation	Sub-Training	Test	Validation	Sub-Training	Test	Validation
ND_Total_^b^	2707	1160	1656	2707	1160	1656	2707	1160	1656
ND_active_^b^	1027	459	620	1027	459	620	1027	459	620
CCD_active_^c^	916	413	553	901	399	541	905	409	546
Sensitivity (%)	89.2	90.0	88.6	87.4	88.6	88.5	88.2	89.9	88.7
ND_inactive_^b^	1680	701	1036	1680	701	1036	1680	1680	1036
CCD_inactive_^c^	1472	626	918	1468	621	917	1481	630	919
Specificity (%)	87.6	89.3	89.2	87.7	86.9	87.3	88.1	89.1	88.1
*F*-measure	0.852	0.872	0.857	0.842	0.851	0.845	0.849	0.871	0.851
Accuracy (%)	88.2	89.6	88.8	87.5	87.9	88.0	88.1	89.6	88.5
MCC ^d^	0.756	0.785	0.767	0.741	0.75	0.749	0.753	0.784	0.758

^a^ LDA classification statistical parameters. ^b^ ND: Number of datapoints. ^c^ Correctly classified datapoints. ^d^ Matthews correlation coefficient.

**Table 3:** Predictive ability by different LDA models.

Classification ^a^	GA-LDA Model			Consensus LDA Model		
	Sub-Training	Test	Validation	Sub-Training	Test	Validation
ND_Total_	2707	1160	1656	2707	1160	1656
ND_active_	1027	459	620	1027	459	620
CCD_active_	916	413	553	902	406	541
Sensitivity (%)	89.19	89.98	88.61	87.83	88.45	87.26
ND_inactive_	1680	701	1036	1680	701	1036
CCD_inactive_	1472	626	918	1486	633	927
Specificity (%)	87.62	89.30	89.19	88.45	90.30	89.48
*F*-measure	0.852	0.872	0.857	0.850	0.870	0.852
Accuracy (%)	88.21	89.57	88.83	88.22	89.57	88.65
MCC	0.756	0.785	0.767	0.754	0.783	0.760

^a^ LDA classification statistical parameters (For information see footnotes on Table 1).

**Table 5:** Hyper-parameters values explored in the machine learning tools applied for development of the non-linear mt-QSAR models.

Method	Parameters Tuned	Parameters Selected	10-Fold CV Accuracy (%) ^a^
RF	Bootstrap: True/False	False	
	Criterion: Gini, Entropy,	Gini	
	Maximum depth: 10, 30, 50, 70, 90, 100, None	90	
	Maximum features: Auto, Sqrt	Sqrt	91.02
	Minimum samples leaf: 1, 2, 4	1	
	Minimum samples split: 2, 5, 10	5	
	Number of estimators: 50, 100, 200,500	200	
k*NN*	Number of neighbors: 1–31	20	
	Weight options: Uniform, Distance	Distance	79.20
	Algorithms: Auto, Ball tree, kd_tree, brute	Auto	
Xgboost	Minimum child weight: 1,5,10	1	
	Gamma: 0, 0.5, 1, 1.5, 2, 5	0	
	Sum sample: 0.6, 0.8, 1.0	0.8	91.54
	Number of estimators: 50, 100, 200,300	100	
	Maximum depth: 3, 4, 5	5	
RBF-SVC	C: 0.1, 1, 10, 100, 1000	1	62.30
	Gamma: 1, 0.1, 0.01, 0.001	1	
MLP	Hidden layer sizes:(50,50,50), (50,100,50), (100,)	(100,)	
	Activation: Identity, Logistic, Tanh, Relu	Relu	
	Solver: SGD, Adam	Adam	82.97
	Alpha: 0.0001, 0.001, 0.01,1	0.0001	
	Learning rate: Constant, Adaptive, Inverse scaling	Adaptive	
DT	Criterion: Gini, EntropyMaximum depth: 10,30,50,70,90,100, NoneMaximum features: Auto, SqrtMinimum samples leaf: 1,2,4Minimum samples split: 2–50	Entropy100Sqrt113	84.33
NB	Alpha: 1,0.5,0.1Fit prior: True, False	0.1True	69.40

**^a^** The cross-validation accuracy was estimated only on the sub-training set.

**Table 6:** Overall predictivity of the RF and Xgboost non-linear models.

Classification ^a^	RF			Xgboost		
	Sub-Training (10-CV)	Test	Validation	Sub-Training (10-CV)	Test	Validation
ND_Total_	2707	1160	1656	2707	1160	1656
ND_active_	1027	459	620	1027	459	620
CCD_active_	919	417	573	932	422	578
Sensitivity (%)	89.48	92.58	93.53	90.75	91.87	93.24
ND_inactive_	1680	701	1036	1680	701	1036
CCD_inactive_	1545	649	969	1546	644	966
Specificity (%)	91.96	90.85	92.42	92.02	91.94	93.23
*F*-measure	0.883	0.899	0.909	0.891	0.900	0.912
Accuracy (%)	91.02	91.90	93.11	91.54	91.90	93.24
MCC	0.810	0.831	0.854	0.822	0.832	0.857

^a^ Non-linear classification statistical parameters (For information see footnotes on Table 1).

**Table 7:** The predictive accuracies of GA-LDA and Xgboost models with respect to the different experimental elements $c_{j}$.

$c_{j}$	$m_{e}$	$a_{t}$	$b_{t}$	Test Set					External Validation Set				
				*N*_Sample_^a^	Xgboost			GA-LDA	*N*_Sample_^a^		Xgboost	GA-LDA	
					# Incorrect ^b^	% Accuracy	# Incorrect ^b^	% Accuracy	# Incorrect ^b^		% Accuracy	# Incorrect ^b^	% Accuracy
**1**	**IC50**	**B**	**AKT**	**509**	80	84.28	95	81.34	707	95	86.56	142	79.92
9	Ki	F	AKT2	163	2	98.77	2	98.77	238	0	100.00	5	97.90
10	Ki	F	AKT3	148	1	99.32	3	97.97	236	2	99.15	2	99.15
8	Ki	F	AKT	156	3	98.08	2	98.72	203	1	99.51	2	99.01
2	IC50	B	AKT2	121	2	98.35	14	88.43	167	8	95.21	23	86.23
3	IC50	B	AKT3	35	4	88.57	4	88.57	53	2	96.23	3	94.34
5	Ki	B	AKT	17	2	88.24	1	94.12	27	2	92.59	3	88.89
6	Ki	B	AKT2	4	0	100.00	0	100.00	12	1	91.67	1	91.67
4	IC50	F	AKT	4	0	100.00	0	100.00	9	1	88.89	3	66.67
7	Ki	B	AKT3	3	0	100.00	0	100.00	4	0	100.00	1	75.00

^a^ Number of data samples. ^b^ Number of data samples (#) incorrectly predicted by the model.

**Table 8:** Number of experimental conditions pertaining to the most potential virtual hits gathered by the GA-LDA and Xgboost models.

Compound	No of Experimental Conditions (GA-LDA)	No of Experimental Conditions (Xgboost)
Asn0019	10	6
Asn0021	10	6
Asn0022	10	6
Asn2706	7	6
Asn5093	8	6
Asn5283	7	6
Asn6236	7	6

**Table 9:** Fitting of the virtual hits to the structure-based pharmacophores predicted by the PharmMapper webserver.

Compound	PDB ID	Target Name	Feature Type ^a^	No of Features	Fit Score
Asn0019	3CQU	AKT1	2H,A,D	4	2.925
Asn0019	2UW9	AKT2	3H,P,A,D	6	3.078
Asn0021	3CQU	AKT1	2H,A,D	4	2.274
Asn0021	2UW9	AKT2	3H,P,A,D	6	3.385
Asn0022	3CQU	AKT1	2H,A,D	4	2.724
Asn0022	2UW9	AKT2	3H,P,A,D	6	3.000
Asn5093	3CQU	AKT1	2H,A,D	4	2.637
Asn5093	2UW9	AKT2	3H,P,A,D	6	3.135
Asn6236	3CQU	AKT1	2H,A,D	4	3.126
Asn6236	2WU9	AKT2	3H,P,A,D	6	2.955

^a^ A: Acceptor, D: Donor, H: Hydrophobic, P: Positive ionizable.

**Table 10:** Calculated binding free energies (∆G_bind_ in kcal/mol) for the AKT1, ATK2, and ATK3 bound ligands.

Compound	AKT1	AKT2	AKT3
Asn0019	−32.54	−27.61	−43.61
Asn0021	−25.81	−29.03	−39.04
Asn0022	−27.75	−23.56	−37.29
Asn5093	−36.34	−29.44	−22.67
Asn6236	−18.08	−19.72	−26.82
GSK690693	−46.88	−29.78	−43.17

## Supplementary Materials

The following are available online https://www.mdpi.com/article/10.3390/ijms22083944/s1, Table S1: Degree of collinearity among the variables of the GA-LDA model; Table S2: Degree of collinearity among the variables of the FS-LDA model; Table S3: Degree of collinearity among the variables of the SFS-LDA model; Figure S1: ROC curves for the two best non-linear models (Xgboost–ROC-AUC score (test): 0.919, ROC-AUC score (validation): 0.932, and RF–AUROC: ROC-AUC score (test): 0.917, ROC-AUC score (validation): 0.930); Table S4: Experimental conditions under which the virtual hits were predicted to be active; Table S5: Molecular docking results with binding energy values (in kcal/mol) of the virtual hits; Figure S2: Fitting of virtual hits to the structure-based pharmacophores of AKT1 (PDB: 3CQU) and AKT2 (PDB: 2UW9) enzymes; Figure S3: Fitting of virtual hits to the structure-based pharmacophores of AKT1 (PDB: 3CQU) and AKT2 (PDB: 2UW9) enzymes.; Figure S4: Protein backbone RMSD, ligand RMSD, RMSF and radius of gyration plots for the AKT1 complexes.; Figure S5: Protein backbone RMSD, ligand RMSD, RMSF and radius of gyration plots for the AKT2 complexes; Figure S6: Protein backbone RMSD, ligand RMSD, RMSF and radius of gyration plots for the AKT3 complexes; Figure S7: (A) Alignment of the AKT3 target sequence with potential template; (B) Z-score estimation of the AKT3 homology model; (C) Local QMEAN estimates after manual refinement; (D) AKT3 homology model built using the Swiss-Model server and the 6CCy.1.A template 3D structure; Figure S8: Ramachandran plots for the homology model of AKT3 enzyme. Starting_protein_ligand_complexes.zip: PDB files containing the complexes of virtual hits with the enzymes, used in the MD simulations.
