# Supplementary material for: AKT Inhibitors: The Road Ahead to Computational Modeling-Guided Discovery
Source: Int J Mol Sci. 2021 Apr 11;22(8):3944. doi: 10.3390/ijms22083944 (PMC8070654; doi:10.3390/ijms22083944)
Supplement: Supplementary file 1 [file ijms-22-03944-s001.pdf]

Table S1. Degree of collinearity among the variables of the GA-LDA model.

Variable	D[Mor27m]a_t	D[Mor21u]a_t	D[C-032]m_e	D[Wi_D/Dt]m_e	D[nArNHR]a_t	D[Mor32m]b_t	D[VE1_Dz(Z)]b_t	D[nRNH2]a_t	D[F02[N-O]]m_e	D[L2m]b_t
D[Mor27m]a_t	1.00	-0.15	0.00	0.05	-0.16	0.55	-0.16	0.04	0.00	0.03
D[Mor21u]a_t	-0.15	1.00	-0.10	-0.63	-0.07	-0.13	-0.57	0.01	-0.16	-0.47
D[C-032]m_e	0.00	-0.10	1.00	0.10	0.41	-0.02	0.08	-0.18	-0.04	0.12
D[Wi_D/Dt]m_e	0.05	-0.63	0.10	1.00	-0.04	-0.08	0.84	0.31	0.42	0.52
D[nArNHR]a_t	-0.16	-0.07	0.41	-0.04	1.00	-0.12	-0.02	-0.21	-0.06	-0.11
D[Mor32m]b_t	0.55	-0.13	-0.02	-0.08	-0.12	1.00	-0.14	0.12	-0.19	0.16
D[VE1_Dz(Z)]b_t	-0.16	-0.57	0.08	0.84	-0.02	-0.14	1.00	0.33	0.30	0.54
D[nRNH2]a_t	0.04	0.01	-0.18	0.31	-0.21	0.12	0.33	1.00	-0.04	0.10
D[F02[N-O]]m_e	0.00	-0.16	-0.04	0.42	-0.06	-0.19	0.30	-0.04	1.00	-0.03
D[L2m]b_t	0.03	-0.47	0.12	0.52	-0.11	0.16	0.54	0.10	-0.03	1.00

Table S2. Degree of collinearity among the variables of the FS-LDA model.

**Variable**	D[nRNH2]a_t	D[L2m]b_t	D[CATS3D_18_DL]b_t	D[D/Dtr05]b_t	D[C-030]b_t	D[nPyridines]m_e	D[nCt]b_t	D[T(N..O)]m_e	D[CATS3D_07_DA]m_e	D[CA
D[nRNH2]a_t	1.00	0.10	0.32	0.16	0.07	0.32	0.30	0.27	0.38	
D[L2m]b_t	0.10	1.00	0.12	0.33	0.32	-0.02	0.39	0.17	0.09	
D[CATS3D_18_DL]b_t	0.32	0.12	1.00	0.04	-0.05	-0.03	0.03	0.61	0.37	
D[D/Dtr05]b_t	0.16	0.33	0.04	1.00	0.07	0.06	0.20	0.19	0.19	
D[C-030]b_t	0.07	0.32	-0.05	0.07	1.00	-0.31	0.29	-0.02	-0.03	
D[nPyridines]m_e	0.32	-0.02	-0.03	0.06	-0.31	1.00	0.02	-0.03	0.03	
D[nCt]b_t	0.30	0.39	0.03	0.20	0.29	0.02	1.00	0.01	0.08	
D[T(N..O)]m_e	0.27	0.17	0.61	0.19	-0.02	-0.03	0.01	1.00	0.58	
D[CATS3D_07_DA]m_e	0.38	0.09	0.37	0.19	-0.03	0.03	0.08	0.58	1.00	
D[CATS3D_10_PL]b_t	0.28	0.04	0.18	0.01	-0.06	0.04	0.10	0.23	0.31	

Table S3. Degree of collinearity among the variables of the SFS-LDA model.

Variable	D[nRNH2]m_e	D[H-052]m_e	D[CATS2D_06_DD]a_t	D[SsNH2]a_t	D[T(N..N)]a_t	D[F07[N-Cl]]a_t	D[Mor31u]b_t	D[CATS2D_02_DD]b_t	D[B03[S-Br]]b_t	D[F08[N-S]]b_t
D[nRNH2]m_e	1.00	0.18	0.43	0.74	0.61	0.02	0.10	0.30	0.16	-0.09
D[H-052]m_e	0.18	1.00	0.24	0.30	0.29	0.03	0.31	0.11	-0.02	-0.06
D[CATS2D_06_DD]a_t	0.43	0.24	1.00	0.34	0.57	0.01	0.19	0.66	-0.01	-0.03
D[SsNH2]a_t	0.74	0.30	0.34	1.00	0.55	-0.06	-0.01	0.25	0.12	-0.08
D[T(N..N)]a_t	0.61	0.29	0.57	0.55	1.00	0.01	0.45	0.53	0.07	-0.01
D[F07[N-Cl]]a_t	0.02	0.03	0.01	-0.06	0.01	1.00	0.08	-0.04	-0.04	-0.02
D[Mor31u]b_t	0.10	0.31	0.19	-0.01	0.45	0.08	1.00	0.05	-0.09	-0.03
D[CATS2D_02_DD]b_t	0.30	0.11	0.66	0.25	0.53	-0.04	0.05	1.00	-0.01	0.00
D[B03[S-Br]]b_t	0.16	-0.02	-0.01	0.12	0.07	-0.04	-0.09	-0.01	1.00	-0.03
D[F08[N-S]]b_t	-0.09	-0.06	-0.03	-0.08	-0.01	-0.02	-0.03	0.00	-0.03	1.00

Table S4. Experimental conditions under which the virtual hits were predicted to be active

**Compound**	Conditions passed (GA-LDA)	Conditions passed (XGBoost)
ASN19	me: IC_{50}, at: B, bt: AKT<br>me: IC_{50}, at: B, bt: AKT2<br>me: IC_{50}, at: B, bt: AKT3<br>me: Ki, at: B, bt: AKT<br>me: Ki, at: B, bt: AKT2<br>me: Ki, at: B, bt: AKT3<br>me: IC_{50}, at: F, bt: AKT<br>me: Ki, at: F, bt: AKT<br>me: Ki, at: F, bt: AKT2<br>me: Ki, at: F, bt: AKT3	me: IC_{50}, at: B, bt: AKT<br>me: IC_{50}, at: B, bt: AKT2<br>me: IC_{50}, at: B, bt: AKT3<br>me: Ki, at: B, bt: AKT<br>me: Ki, at: B, bt: AKT2<br>me: Ki, at: B, bt: AKT3
ASN21	me: IC_{50}, at: B, bt: AKT<br>me: IC_{50}, at: B, bt: AKT2<br>me: IC_{50}, at: B, bt: AKT3<br>me: Ki, at: B, bt: AKT<br>me: Ki, at: B, bt: AKT2<br>me: Ki, at: B, bt: AKT3<br>me: IC_{50}, at: F, bt: AKT<br>me: Ki, at: F, bt: AKT<br>me: Ki, at: F, bt: AKT2<br>me: Ki, at: F, bt: AKT3	me: IC_{50}, at: B, bt: AKT<br>me: IC_{50}, at: B, bt: AKT2<br>me: IC_{50}, at: B, bt: AKT3<br>me: Ki, at: B, bt: AKT<br>me: Ki, at: B, bt: AKT2<br>me: Ki, at: B, bt: AKT3
ASN22	me: IC_{50}, at: B, bt: AKT<br>me: IC_{50}, at: B, bt: AKT2<br>me: IC_{50}, at: B, bt: AKT3<br>me: Ki, at: B, bt: AKT<br>me: Ki, at: B, bt: AKT2<br>me: Ki, at: B, bt: AKT3<br>me: IC_{50}, at: F, bt: AKT<br>me: Ki, at: F, bt: AKT<br>me: Ki, at: F, bt: AKT2<br>me: Ki, at: F, bt: AKT3	me: IC_{50}, at: B, bt: AKT<br>me: IC_{50}, at: B, bt: AKT2<br>me: IC_{50}, at: B, bt: AKT3<br>me: Ki, at: B, bt: AKT<br>me: Ki, at: B, bt: AKT2<br>me: Ki, at: B, bt: AKT3
ASN2706	me: IC_{50}, at: B, bt: AKT<br>me: IC_{50}, at: B, bt: AKT2<br>me: IC_{50}, at: B, bt: AKT3<br>me: Ki, at: B, bt: AKT<br>me: Ki, at: B, bt: AKT2<br>me: Ki, at: B, bt: AKT3<br>me: Ki, at: F, bt: AKT2	me: IC_{50}, at: B, bt: AKT<br>me: IC_{50}, at: B, bt: AKT2<br>me: IC_{50}, at: B, bt: AKT3<br>me: Ki, at: B, bt: AKT<br>me: Ki, at: B, bt: AKT2<br>me: Ki, at: B, bt: AKT3
ASN5093	me: IC_{50}, at: B, bt: AKT<br>me: IC_{50}, at: B, bt: AKT2<br>me: IC_{50}, at: B, bt: AKT3<br>me: Ki, at: B, bt: AKT<br>me: Ki, at: B, bt: AKT2<br>me: Ki, at: B, bt: AKT3<br>me: Ki, at: F, bt: AKT2<br>me: Ki, at: F, bt: AKT3	me: IC_{50}, at: B, bt: AKT<br>me: IC_{50}, at: B, bt: AKT2<br>me: IC_{50}, at: B, bt: AKT3<br>me: Ki, at: B, bt: AKT<br>me: Ki, at: B, bt: AKT2<br>me: Ki, at: B, bt: AKT3
Asn5283	me: IC_{50}, at: B, bt: AKT<br>me: IC_{50}, at: B, bt: AKT2<br>me: IC_{50}, at: B, bt: AKT3<br>me: Ki, at: B, bt: AKT<br>me: Ki, at: B, bt: AKT2<br>me: Ki, at: B, bt: AKT3<br>me: Ki, at: F, bt: AKT2	me: IC_{50}, at: B, bt: AKT<br>me: IC_{50}, at: B, bt: AKT2<br>me: IC_{50}, at: B, bt: AKT3<br>me: Ki, at: B, bt: AKT<br>me: Ki, at: B, bt: AKT2<br>me: Ki, at: B, bt: AKT3
Asn6236	me: IC_{50}, at: B, bt: AKT<br>me: IC_{50}, at: B, bt: AKT2<br>me: IC_{50}, at: B, bt: AKT3<br>me: Ki, at: B, bt: AKT<br>me: Ki, at: B, bt: AKT2<br>me: Ki, at: B, bt: AKT3<br>me: Ki, at: F, bt: AKT2	me: IC_{50}, at: B, bt: AKT<br>me: IC_{50}, at: B, bt: AKT2<br>me: IC_{50}, at: B, bt: AKT3<br>me: Ki, at: B, bt: AKT<br>me: Ki, at: B, bt: AKT2<br>me: Ki, at: B, bt: AKT3

Table S5. Molecular docking results with binding energy values (in kcal/mol) of the virtual hits.

Compound	AKT1		AKT2		AKT3	
	Vina	Autodock	Vina	Autodock	Vina	Autodock
Asn0019	−10.00	−10.34	−9.60	−8.57	−8.40	−8.30
Asn0021	−9.00	−9.04	−9.40	−8.37	−8.40	−8.06
Asn0022	−10.20	−10.10	−9.40	−8.47	−8.50	−8.34
Asn5093	−9.20	−8.28	−7.10	−7.22	−7.60	−7.01
Asn6236	−10.40	−10.47	-9.60	−9.27	−8.80	−8.32
GSK690693	nd [a]	−8.13	nd [a]	−8.27	nd [a]	−10.20

[a] Not determined.

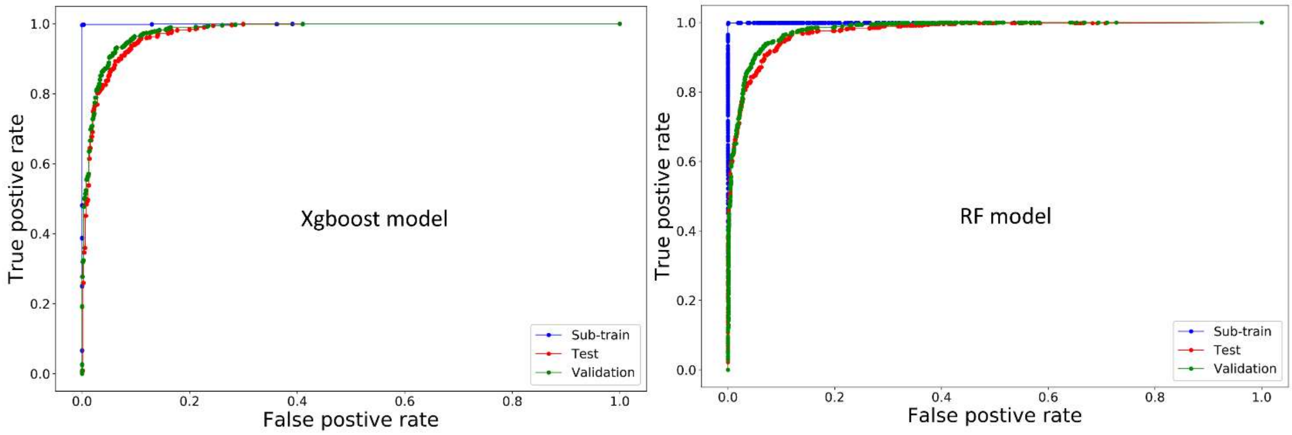

Figure S1. ROC curves for the two best non-linear models (Xgboost – ROC-AUC score (test): 0.919, ROC-AUC score (validation): 0.932, and RF – AUROC: ROC-AUC score (test): 0.917, ROC-AUC score (validation): 0.930)

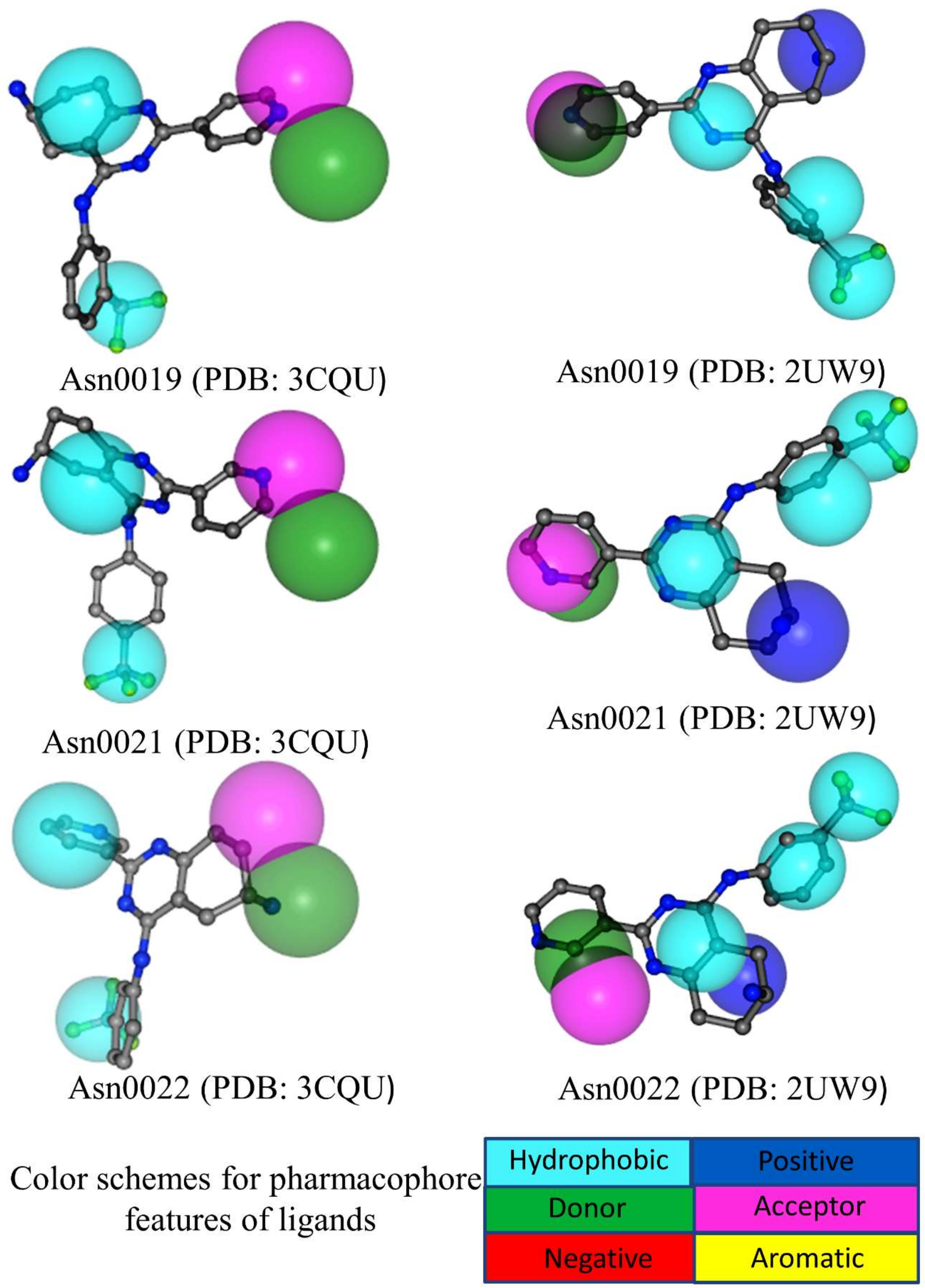

Figure S2. Fitting of virtual hits on the structure-based pharmacophores of AKT1 (PDB: 3CQU) and AKT2 (PDB: 2UW9) enzymes. All

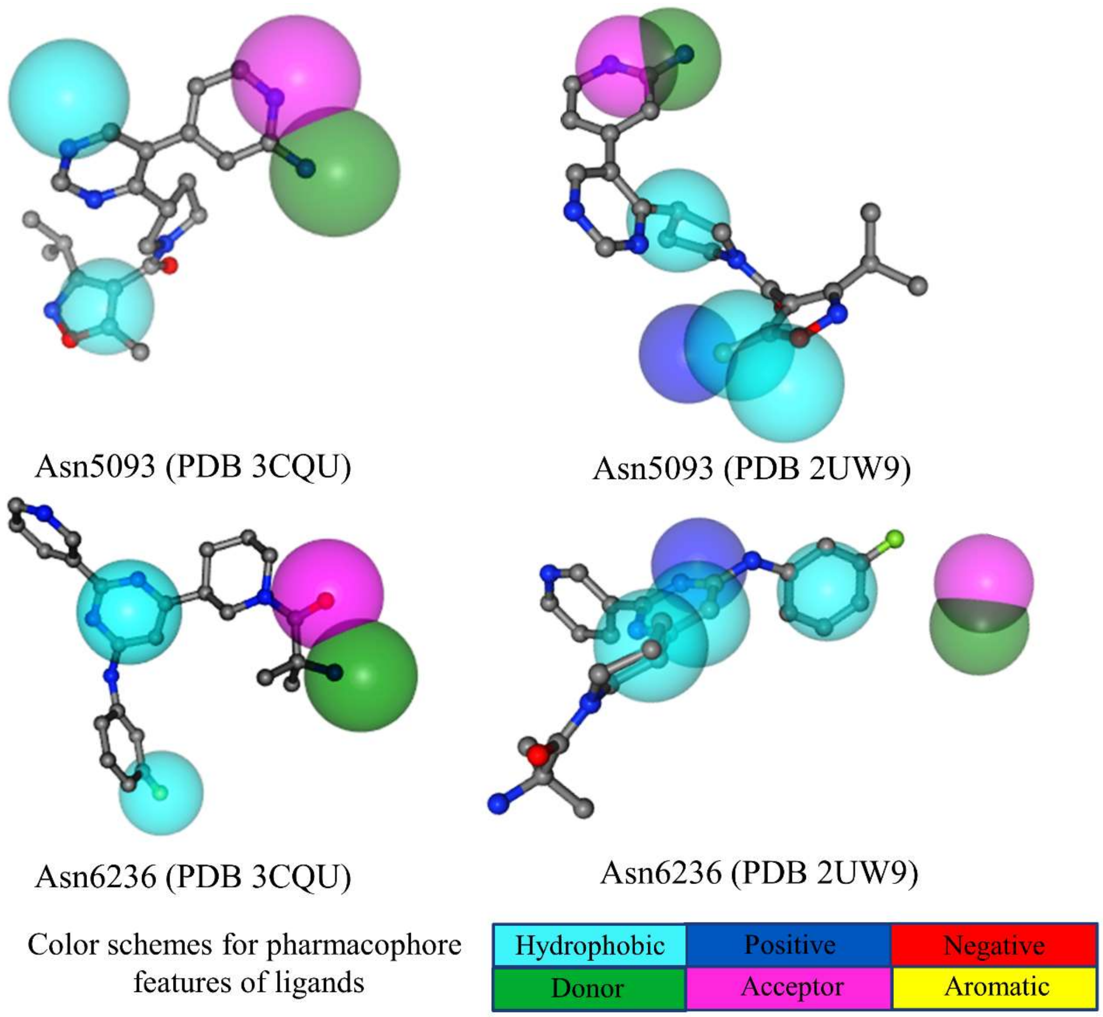

Figure S3. Fitting of virtual hits on the structure-based pharmacophores of AKT1 (PDB: 3CQU) and AKT2 (PDB: 2UW9) enzymes

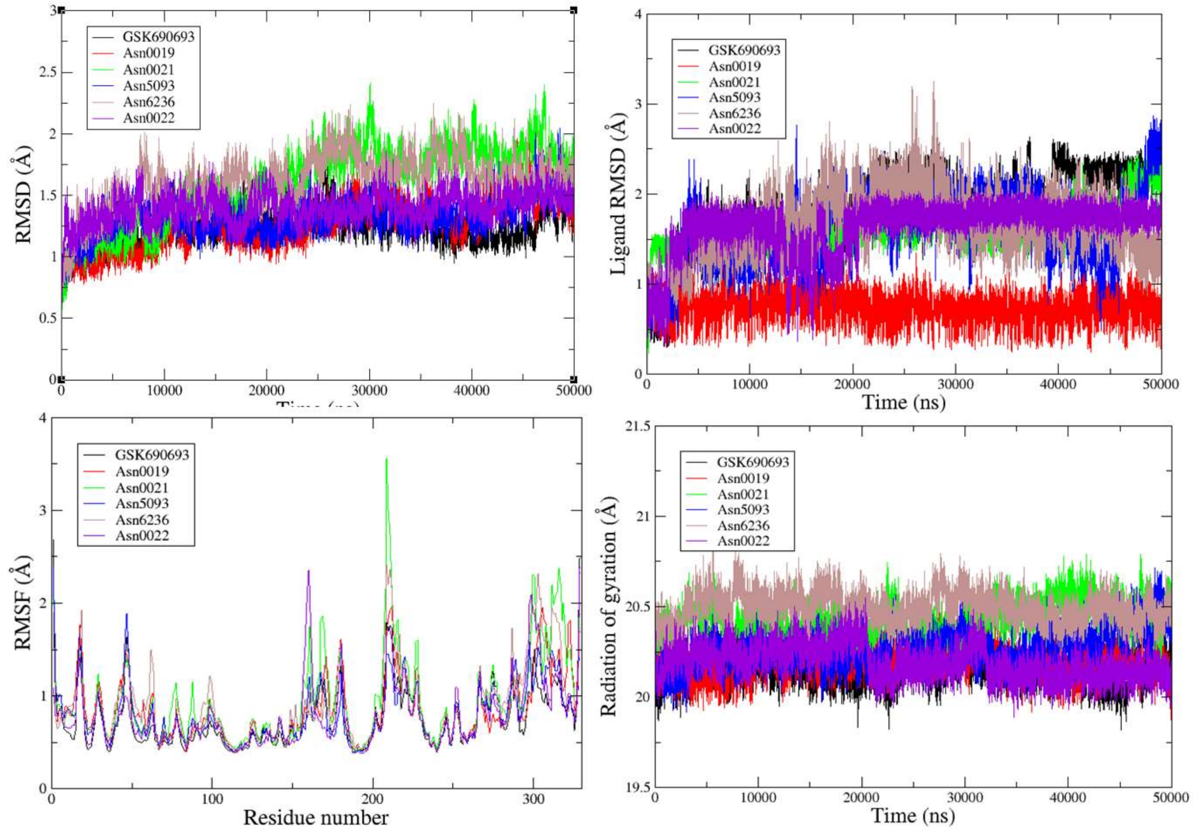

Figure S4. Protein backbone RMSD, ligand RMSD, RMSF and radiation of gyration plots of AKT1 complexes.

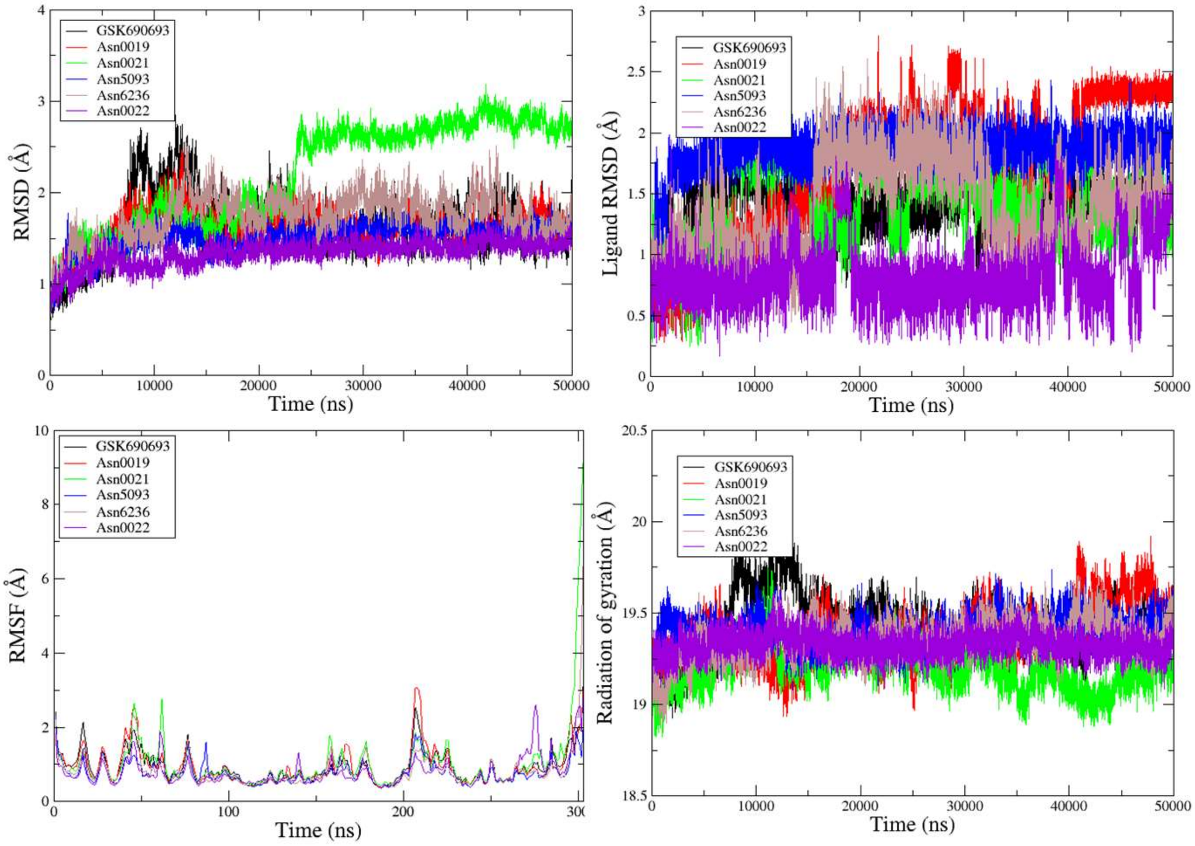

Figure S5. Protein backbone RMSD, ligand RMSD, RMSF and radiation of gyration plots of AKT2 complexes.

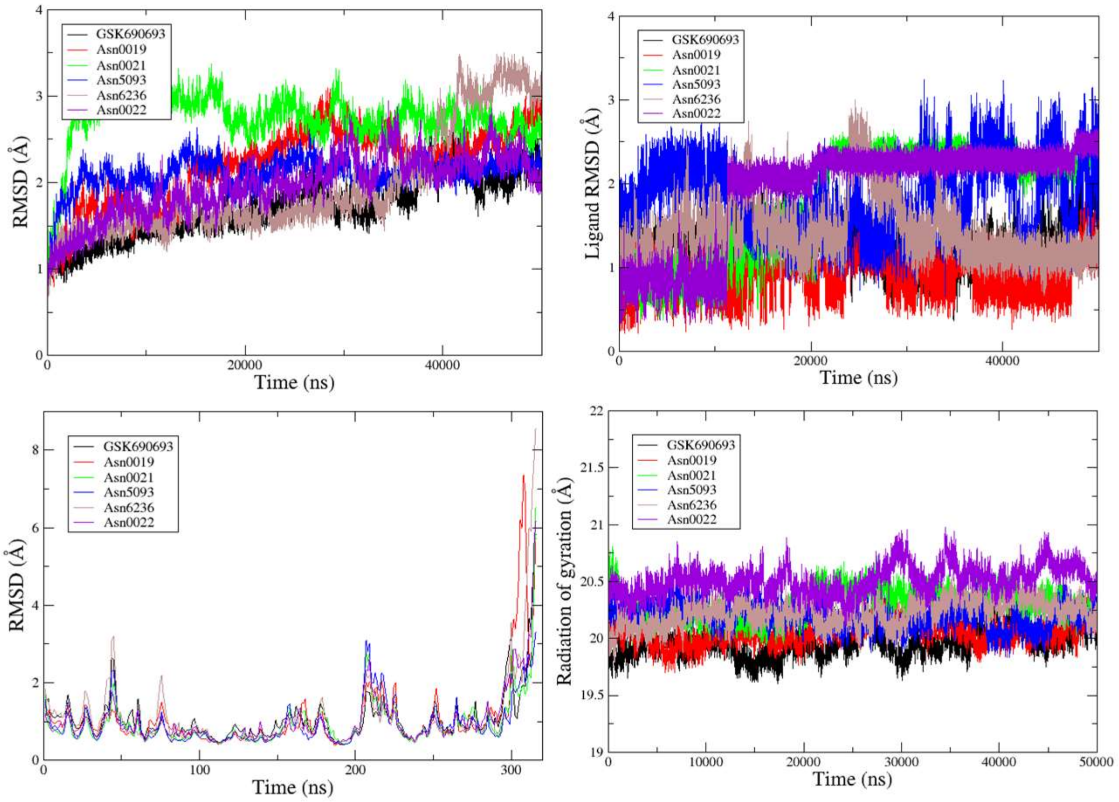

Figure S6. Protein backbone RMSD, ligand RMSD, RMSF and radiation of gyration plots of AKT3 complexes.

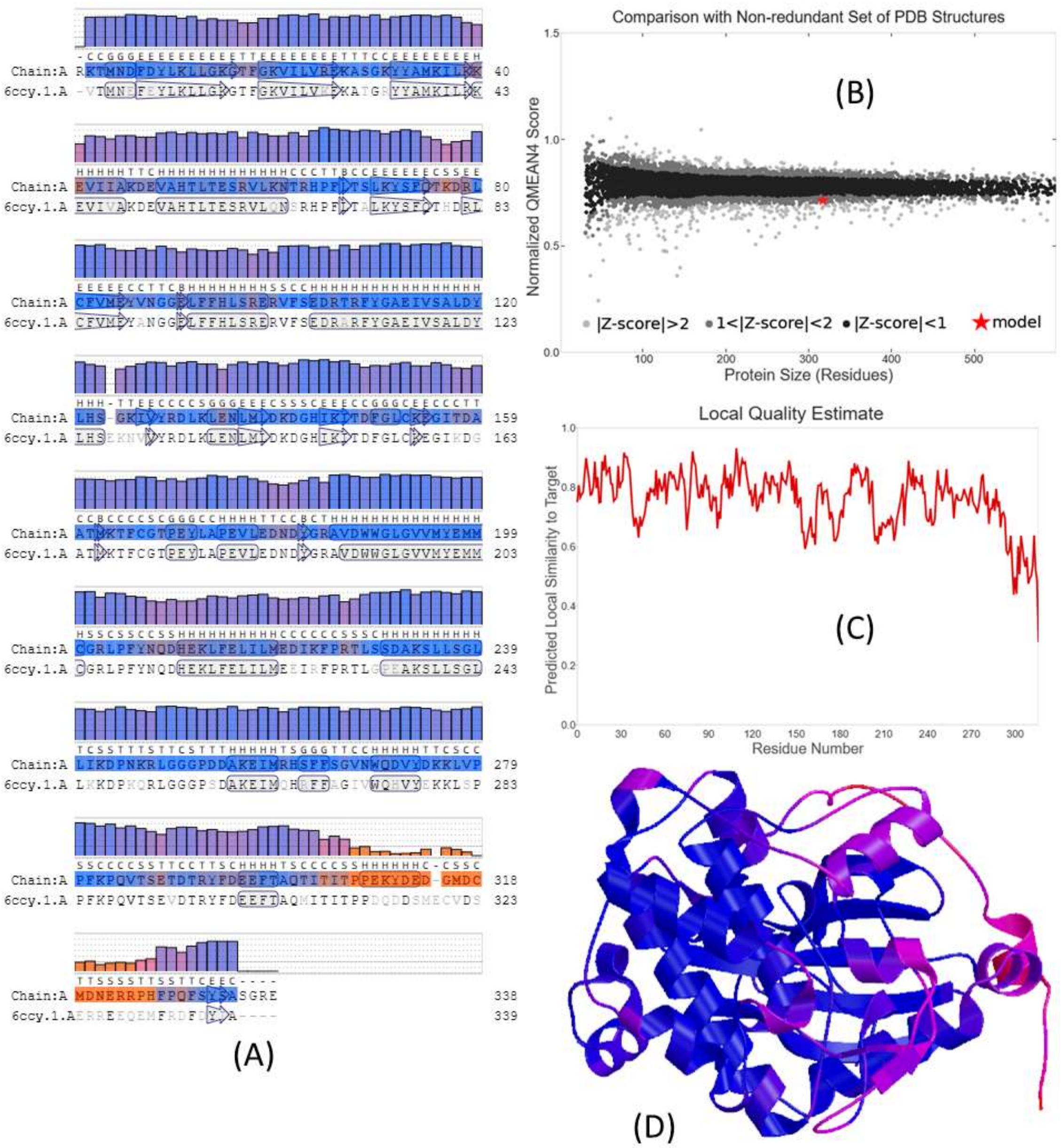

Figure S7. (A) Alignment of AKT3 target sequence with potential template, (B) Z-score estimation of the AKT3 homology model, (C) local QMEAN estimates after manual refinement, (D) AKT3 homology model built using Swiss-Model server using the 6CCy.1.A template 3D structure

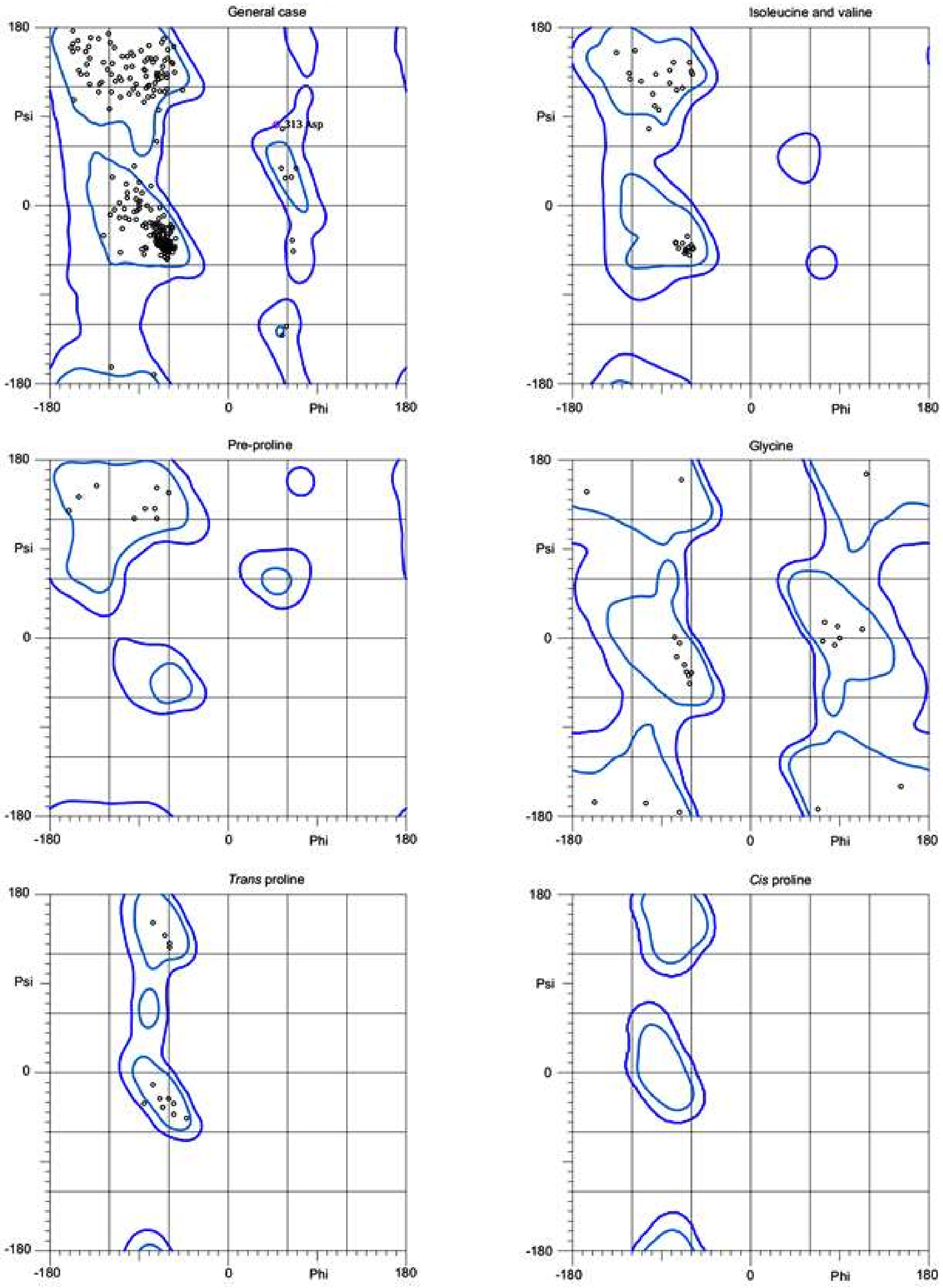

94.6% (298/315) of all residues were in favored (98%) regions.
99.7% (314/315) of all residues were in allowed (>99.8%) regions.

There were 1 outliers (phi, psi):
313 Asp (49.1, 82.5)

Figure S8. Ramachandran plots of the homology model of AKT3 enzyme
